# Racial Disparities in Length of Stay Among Severely Ill Patients Presenting With Sepsis and Acute Respiratory Failure

**DOI:** 10.1001/jamanetworkopen.2023.9739

**Published:** 2023-05-08

**Authors:** Christopher F. Chesley, Marzana Chowdhury, Dylan S. Small, Douglas Schaubel, Vincent X. Liu, Meghan B. Lane-Fall, Scott D. Halpern, George L. Anesi

**Affiliations:** 1 Division of Pulmonary, Allergy, and Critical Care, Department of Medicine, University of Pennsylvania Perelman School of Medicine, Philadelphia; 2 Palliative and Advanced Illness Research Center, University of Pennsylvania Perelman School of Medicine, Philadelphia; 3 Leonard Davis Institute of Health Economics, University of Pennsylvania, Philadelphia; 4 Wharton Department of Statistics and Data Science, University of Pennsylvania, Philadelphia; 5 Department of Biostatistics, Epidemiology, and Informatics, University of Pennsylvania Perelman School of Medicine, Philadelphia; 6 Division of Research, Kaiser Permanente, Oakland, California; 7 Department of Anesthesiology and Critical Care, University of Pennsylvania Perelman School of Medicine, Philadelphia; 8 Department of Medical Ethics and Health Policy, University of Pennsylvania Perelman School of Medicine, Philadelphia

## Abstract

**Importance:**

Although racial and ethnic minority patients with sepsis and acute respiratory failure (ARF) experience worse outcomes, how patient presentation characteristics, processes of care, and hospital resource delivery are associated with outcomes is not well understood.

**Objective:**

To measure disparities in hospital length of stay (LOS) among patients at high risk of adverse outcomes who present with sepsis and/or ARF and do not immediately require life support and to quantify associations with patient- and hospital-level factors.

**Design, Setting, and Participants:**

This matched retrospective cohort study used electronic health record data from 27 acute care teaching and community hospitals across the Philadelphia metropolitan and northern California areas between January 1, 2013, and December 31, 2018. Matching analyses were performed between June 1 and July 31, 2022. The study included 102 362 adult patients who met clinical criteria for sepsis (n = 84 685) or ARF (n = 42 008) with a high risk of death at the time of presentation to the emergency department but without an immediate requirement for invasive life support.

**Exposures:**

Racial or ethnic minority self-identification.

**Main Outcomes and Measures:**

Hospital LOS, defined as the time from hospital admission to the time of discharge or inpatient death. Matches were stratified by racial and ethnic minority patient identity, comparing Asian and Pacific Islander patients, Black patients, Hispanic patients, and multiracial patients with White patients in stratified analyses.

**Results:**

Among 102 362 patients, the median (IQR) age was 76 (65-85) years; 51.5% were male. A total of 10.2% of patients self-identified as Asian American or Pacific Islander, 13.7% as Black, 9.7% as Hispanic, 60.7% as White, and 5.7% as multiracial. After matching racial and ethnic minority patients to White patients on clinical presentation characteristics, hospital capacity strain, initial intensive care unit admission, and the occurrence of inpatient death, Black patients experienced longer LOS relative to White patients in fully adjusted matches (sepsis: 1.26 [95% CI, 0.68-1.84] days; ARF: 0.97 [95% CI, 0.05-1.89] days). Length of stay was shorter among Asian American and Pacific Islander patients with ARF (−0.61 [95% CI, −0.88 to −0.34] days) and Hispanic patients with sepsis (−0.22 [95% CI, −0.39 to −0.05] days) or ARF (−0.47 [−0.73 to −0.20] days).

**Conclusions and Relevance:**

In this cohort study, Black patients with severe illness who presented with sepsis and/or ARF experienced longer LOS than White patients. Hispanic patients with sepsis and Asian American and Pacific Islander and Hispanic patients with ARF both experienced shorter LOS. Because matched differences were independent of commonly implicated clinical presentation–related factors associated with disparities, identification of additional mechanisms that underlie these disparities is warranted.

## Key Points

**Question:**

Do conventionally understood mechanisms of disparities adequately explain differences in hospital length of stay (LOS) between racial and ethnic minority and nonminority groups?

**Findings:**

In this cohort study of 102 362 adult patients with sepsis and/or acute respiratory failure (ARF), Black patients with sepsis and/or ARF had a hospital LOS that was 0.97 to 1.26 days longer than White patients; in contrast, Hispanic patients with sepsis and ARF and Asian American and Pacific Islander patients with ARF were found to have a shorter hospital LOS than White patients. These findings were independent of the factors associated with disparities that were assessed.

**Meaning:**

These findings suggest that commonly understood factors associated with disparities related to clinical presentation and care in the intensive care unit are insufficient to account for racial and ethnic disparities in sepsis and ARF outcomes.

## Teaser

This cohort study uses electronic health record data to measure racial and ethnic disparities in hospital length of stay and assess the associations of patient- and hospital-level factors with length of stay among adult patients at high risk presenting with sepsis or acute respiratory failure.

## Introduction

Patients who identify as belonging to racial and ethnic minority groups, especially those who identify as Black, experience the highest burden of disease^1,2,3,4^ and death^5,6,7,8,9^ from sepsis and acute respiratory failure (ARF). Most large-scale studies have identified a mix of factors, including disease severity, baseline comorbidities,^10,11,12,13^ and between-hospital differences,^2,5,13,14,15,16,17^ that account for these disparities. However, administrative data sets commonly used for these investigations may have difficulty measuring time-varying within-hospital characteristics. Among them, capacity strain (the resource limitation that occurs during routine health care systems operations^18^) alters intensive care unit (ICU) resource delivery.^19,20^ However, the association between capacity strain and disparate clinical outcomes has not been explored. Although a previous study^21^ found that capacity strain did not restrict ICU admission of racial and ethnic minority patients, its association with equitable clinical outcomes (irrespective of ICU triage) has not been studied. In addition, because different patterns in ICU care have been reported for racial and ethnic minority patients,^21^ whether initial ICU admission is associated with inequitable outcomes warrants further investigation. Because initial ICU triage has been associated with (1) longer hospital length of stay (LOS) among patients presenting with sepsis and (2) shorter LOS among patients presenting with ARF,^22^ it is plausible that different rates of ICU admission may impact the equitability of outcomes.

Our objectives in this study were to (1) measure racial and ethnic group–specific LOS differences in a cohort of patients with severe disease (eg, at high risk for adverse outcomes despite not needing immediate life support) who presented with sepsis and/or ARF and (2) quantify the relative associations of demographic characteristics not related to race and ethnicity, disease severity, initial ICU admission, and capacity strain with hospital LOS. We hypothesized that racial and ethnic minority patients would experience the longest LOS, which would be accounted for by matching patients on disease severity and initial ICU admission status.

## Methods

This matched retrospective cohort study used electronic health record (EHR) data from 27 acute care teaching and community hospitals across the Philadelphia metropolitan and northern California areas between January 1, 2013, and December 31, 2018.^19,21,22,23^ Matching analyses were performed between June 1 and July 31, 2022. All hospitals were located in areas with a population greater than 1 million, and 2 hospitals were considered primary university teaching hospitals; the range of inpatient beds was between 50 and 776. This study was approved by the institutional review boards of the study health care systems. A waiver of informed consent was granted because this was a noninterventional study with low or no risk to participants and because the sample size was not feasible for individual consent. The study followed the Strengthening the Reporting of Observational Studies in Epidemiology (STROBE) reporting guideline for cohort studies.^24^

### Study Population

The study population comprised 102 362 patients who were 18 years or older, admitted to either a medical or medical-surgical ward or an ICU through the emergency department (ED), and met criteria for sepsis or ARF based on clinical factors and initial treatment delivery during ED presentation. Criteria for sepsis required an order for antimicrobial medication and microbiological cultures and evidence of acute organ dysfunction adapted from the Third International Consensus Definitions for Sepsis and Septic Shock criteria by the time of admission.^19,23,25,26,27^ Criteria for ARF required evidence of hypoxemia while receiving supplemental oxygen or clinically relevant hypercarbia and/or hypoxia.^19,23,27,28^ Consistent with previous studies,^19,20,21,22^ we enrolled patients with a Laboratory Acute Physiology Score, version 2 (LAPS2; score range, 0-414, with higher scores indicating greater physiological instability), of 100 or higher who did not receive mechanical ventilation or vasopressors in the ED. These criteria were selected to identify a population of patients who were at high risk for death or longer LOS but who had an admission decision (eg, general ward or ICU) that was plausibly at clinician discretion.^22,29^ Patients were excluded if they were receiving hospice care or had an order for receipt of only comfort measures during ED presentation.

### Study Variables

Exposure variables were patient-identified race and ethnicity. If unable to self-identify their race and ethnicity, designation by appointed decision-makers occurred. If unavailable, race and/or ethnicity was designated by hospital staff. Racial and ethnic categories were defined as Hispanic, non-Hispanic Asian American and Pacific Islander (hereinafter, Asian or Pacific Islander), non-Hispanic Black (hereinafter, Black), non-Hispanic White (hereinafter, White), and non-Hispanic multiracial (hereafter, multiracial).^30^ Matching variables were patient age, sex (defined as female or male based on study hospital reporting during the study period), insurance type, do not resuscitate (DNR) code status (determined based on presence of a DNR order while in the ED or up to 3 hours after hospital admission), disease severity (measured using the LAPS2 and the Comorbidity Point Score, version 2 [COPS2]; score range, 0-1014, with higher scores indicating higher burden of coexisting conditions),^23,31,32,33^ and a hospital-wide capacity strain index. The Capacity Strain Index, a validated measure of capacity strain throughout a given study hospital,^19,28^ was derived from 22 factors measuring resource limitation due to census occupancy, disease acuity, and turnover across hospital wards, ICUs, and stepdown units. The capacity strain index is an empirically derived measurement that theoretically has no bounds but is centered around a mean value of 0; higher values indicate greater hospital-wide capacity strain. Indices were developed at the hospital level using a previous approach^21^ in which patient race and ethnicity were not used as estimation variables. The primary outcome was hospital LOS, defined as the time of hospital admission to the time of patient discharge or inpatient death.

### Statistical Analysis

Descriptive statistics were calculated to illustrate hypothesized baseline covariate imbalances that motivated our matching approach. Due to skewed distributions, the Kruskal-Wallis test was used for continuous variables; the Pearson χ^2^ test was used for categorical variables.

#### Matching Strategy

Due to hypothesized covariate imbalances between study groups, we performed nearest-neighbor matching, which quantifies average outcome differences between 2 alternative exposure groups, using a with-replacement strategy. Participants with alternative exposures (eg, different racial or ethnic identities) were selected with replacement to minimize Mahalanobis covariate distances between matched pairs; mean outcome differences between groups were then determined.^34,35^ Our analyses considered racial and ethnic minority identities and White identities as alternative exposure groups and were stratified by specific racial and ethnic minority identity. Patients with White identity were chosen as the reference group due to hypothesized favorable clinical outcomes. In addition, based on the recommendation of other researchers,^36,37^ we additionally matched on propensity scores that modeled patient racial and ethnic identity to further reduce potential biases. Matches selected participants of alternative groups on a 1:2 ratio. Covariate balance after matching was assessed using SD differences.^38,39,40,41^ An SD lower than 0.20 was considered sufficient balance,^42^ and an SD lower than 0.10 was considered ideal.^41^

**Figure 1.**
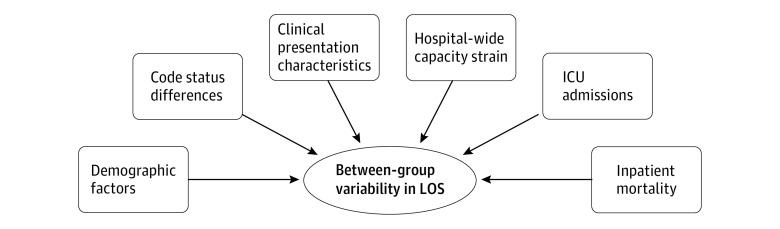
Conceptual Framework of Factors Associated With Between-Group Variability in Hospital Length of Stay (LOS) Factors associated with variability were hypothesized based on a review of previous literature.^2,5,13,14,15,16,17^ ICU indicates intensive care unit.

We hypothesized that patient- and treatment-level characteristics would be associated with between-group differences (Figure 1) based on review of previous literature.^2,5,13,14,15,16,17^ Our primary analysis accounted for these possible associated factors by matching across all domains. Because all matched study variables were included in the inpatient death–matched analysis (eTable 1 in Supplement 1), it was considered the primary analysis.

#### Secondary Analysis

For the secondary analysis, we adapted exterior matching techniques, which comprise significance testing of the variables that define separate matches based on the same exposure group.^41,42,43^ This testing is accomplished by comparing the 2 matched control groups developed from the single exposed group. For example, to determine the statistical importance of matching on capacity strain for patients who identify as Black, Black patients would be matched to White patients on presentation characteristics. In a separate match, Black patients would be additionally matched to White patients on capacity strain. An exterior match would then compare LOS between (1) White patients matched to Black patients based on clinical presentation characteristics and (2) White patients matched to Black patients based on clinical presentation and capacity strain. The matched LOS difference between the 2 groups of White patients provides an estimate of the significance of additionally matching on capacity strain. Because, to our knowledge, the principles of exterior matching have not previously been adapted to a with-replacement matching approach, we referred to the technique as implicit matching. For completeness, we calculated LOS differences based on 6 distinct matches formed by the stepwise addition of the following matching variables: demographic characteristics not related to race and ethnicity, DNR code status, clinical presentation, capacity strain, initial ICU admission, and inpatient death (eTable 1 in Supplement 1). Implicit matching was performed if LOS differences between the matches of clinical presentation, capacity strain, initial ICU admission, or inpatient death differed by 10% or more. Further discussion of the matching strategy is available in the eMethods in Supplement 1.

#### Sensitivity Analyses

Between-group differences in mortality may bias LOS estimates. Therefore, in sensitivity analyses, we first accounted for this bias by comparing the matches of initial ICU admission and inpatient death. In addition, we performed placement of death and survivor average causal effects (SACE) analyses. Placement of death describes a composite outcome equivalent to a patient’s hospital LOS if discharged from the hospital; however, if inpatient death or hospice discharge occurs, LOS is equivalent to the 95th percentile of the population distribution (ie, a long and undesirable LOS).^22,44^ The placement of death approach has been used in multiple analyses that analyze LOS in study populations with high mortality rates.^19,22,45^ Because we hypothesized that LOS would differ based on patient group identity, LOS was assigned to the 95th percentile specific to patient race, ethnicity, and disease. Matches of death-placed LOS were determined using the matching strategy described in the previous paragraph.

Survivor average causal effects analyses estimate treatment effects among the theoretical subpopulation of patients who would have always survived (ie, always survivors) regardless of exposure identity. We adapted a previously developed method that applied inverse probability weighting to estimate the probability of being an always survivor.^46^ We applied the weighting technique to marginal structural models that were quantile regression models of the LOS of race-, ethnicity-, and disease-specific differences in median LOS. Covariates included matching variables from the match of inpatient death, including study hospital as a fixed effect.^47^ The SACE regression estimates can be interpreted as between-group differences in median LOS among patients estimated to always survive.

The statistical significance for all 2-sided hypothesis tests was set at 2-tailed *P* < .05. Analyses were performed using Stata software, version 15.1 (StataCorp, LLC).

## Results

### Study Population Characteristics

Among 102 362 patients, the median (IQR) age was 76 (65-85) years; 52 705 (51.5%) were male, 49 657 (48.5%) were female, and 10 454 (10.2%) self-identified as Asian or Pacific Islander, 13 994 (13.7%) as Black, 9907 (9.7%) as Hispanic, 62 139 (60.7%) as White, and 5868 (5.7%) as multiracial. Of those, 84 685 patients had sepsis and 42 008 had ARF. Overall, Hispanic patients had the lowest COPS2 (median [IQR], 102 [57-142]) compared with Asian or Pacific Islander patients (median [IQR], 103 [57-144]), White patients (median [IQR], 109 [69-147]), Black patients (median [IQR], 115 [65-162]), and multiracial patients (median [IQR], 117 [80-153]) patients. Across the entire study population, insurance coverage varied by race and ethnicity. Private insurance was common among Asian or Pacific Islander patients (8819 [84.4%]), Hispanic patients (7864 [79.4%]), White patients (49 043 [78.9%]), and multiracial patients (5394 [91.9%]) patients but was less common among Black patients (7528 [53.8%]). Medicare coverage was most common among Black patients (3160 [22.6%]) and less common among Asian or Pacific Islander patients (174 [1.7%]), Hispanic patients (586 [5.9%]), White patients (8036 [12.9%]), and multiracial patients (140 [2.4%]) patients. Medicaid coverage was also most common among Black patients (1492 [10.7%]) and less common among Asian or Pacific Islander patients (240 [2.3%]), Hispanic patients (432 [4.4%]), White patients (1238 [2.0%]), and multiracial patients (148 [2.5%]).

Among patients with sepsis, the median (IQR) age was 76 (65-85) years; 8957 patients (10.6%) identified as Asian or Pacific Islander, 10 586 (12.5%) as Black, 8444 (10.0%) as Hispanic, 51 749 (61.1%) as White, and 4949 (5.8%) as multiracial. Among patients with ARF, the median (IQR) age was 75 (64-84) years; 3982 patients (9.5%) identified as Asian or Pacific Islander, 6425 (15.3%) as Black, 3422 (8.1%) as Hispanic, 25 840 (61.5%) as White, and 2339 (5.6%) as multiracial (Table 1). All groups differed significantly on all baseline characteristics (*P* < .001 for all comparisons). No missing data were observed.

**Table 1.** Characteristics of Study Population

Characteristic	Patients, No. (%)											
	Sepsis						Acute respiratory failure					
	Total (N = 84 685)	Race or ethnicity^a^					Total (N = 42 008)	Race or ethnicity^a^				
		Asian American and Pacific Islander (n = 8957)	Black (n = 10 586)	Hispanic (n = 8444)	White (n = 51 749)	Multiracial (n = 4949)		Asian American and Pacific Islander (n = 3982)	Black (n = 6425)	Hispanic (n = 3422)	White (n = 25 840)	Multiracial (n = 2339)
Age, median (IQR), y	76 (65-85)	77 (66-85)	69 (58-80)	73 (59-83)	77 (67-86)	79 (69-86)	75 (64-84)	76 (66-85)	67 (57-77)	72 (60-82)	76 (67-85)	77 (68-85)
Sex												
Male	44 210 (52.2)	5024 (56.1)	5254 (49.6)	4606 (54.5)	26 914 (52.0)	2412 (48.7)	20 598 (49.0)	2258 (56.7)	2954 (46.0)	1799 (52.6)	12 552 (48.6)	1035 (44.2)
Female	40 475 (47.8)	3933 (43.9)	5332 (50.4)	3838 (45.5)	24 835 (48.0)	2537 (51.3)	21 410 (51.0)	1724 (43.3)	3471 (54.0)	1623 (47.4)	13 288 (51.4)	1304 (55.8)
Insurance type												
Private	66 658 (78.7)	7626 (85.1)	6014 (56.8)	6780 (80.3)	41 670 (80.5)	4568 (92.3)	30 737 (73.2)	3240 (81.4)	3110 (48.4)	2601 (76.0)	19 658 (76.1)	2128 (91.0)
Medicare	8951 (10.6)	148 (1.7)	2176 (20.6)	464 (5.5)	6050 (11.7)	113 (2.3)	5636 (13.4)	68 (1.7)	1637 (25.5)	220 (6.4)	3657 (14.2)	54 (2.3)
Medicaid	2722 (3.2)	201 (2.2)	1072 (10.1)	345 (4.1)	985 (1.9)	119 (2.4)	1636 (3.9)	102 (2.6)	711 (11.1)	182 (5.3)	566 (2.2)	75 (3.2)
Unknown	6354 (7.5)	982 (11.0)	1324 (12.5)	855 (10.1)	3044 (5.9)	149 (3.0)	3999 (9.5)	572 (14.4)	967 (15.1)	419 (12.2)	1959 (7.6)	82 (3.5)
DNR order	27 340 (32.3)	2560 (28.6)	1785 (16.9)	2069 (24.5)	19 205 (37.1)	1721 (34.8)	13 090 (31.2)	1132 (28.4)	894 (13.9)	863 (25.2)	9443 (36.5)	758 (32.4)
LAPS2, median (IQR)^b^	124 (111-143)	125 (112-145)	125 (111-145)	124 (111-142)	124 (111-143)	126 (111-145)	126 (112-146)	129 (113-151)	123 (110-143)	125 (112-146)	127 (112-146)	129 (113-149)
COPS2, median (IQR)^c^	108 (65-149)	103 (57-144)	116 (64-165)	100 (56-142)	108 (67-148)	117 (78-153)	116 (78-153)	111 (67-149)	117 (68-159)	110 (68-147)	117 (79-152)	123 (93-157)
Initial admission to ICU	16 535 (19.5)	1821 (20.3)	2830 (26.7)	1713 (20.3)	9261 (17.9)	910 (18.4)	11 852 (28.2)	1291 (32.4)	2346 (36.5)	1054 (30.8)	6519 (25.2)	642 (27.4)
Inpatient death	14 379 (17.0)	1488 (16.6)	1454 (13.7)	1207 (14.3)	9378 (18.1)	852 (17.2)	8395 (20.0)	825 (20.7)	763 (11.9)	611 (17.9)	5713 (22.1)	483 (20.6)
Hospital LOS, median (IQR), d	3.9 (2.3-6.7)	3.7 (2.2-6.2)	4.7 (2.7-8.1)	3.7 (2.2-6.1)	3.8 (2.3-6.5)	3.7 (2.1-6.2)	3.9 (2.1-6.7)	3.6 (2.0-6.4)	4.1 (2.2-7.5)	3.7 (2.0-6.3)	3.9 (2.2-6.7)	3.8 (2.1-6.6)

Abbreviations: COPS2, Comorbidity Point Score, version 2; DNR, do not resuscitate; ICU, intensive care unit; LAPS2, Laboratory Acute Physiology Score, version 2.

^a^ *P* < .001 for all comparisons between race and ethnicity categories.

^b^ Score range, 0 to 414, with higher scores indicating greater physiological instability.

^c^ Score range, 0 to 1014, with higher scores indicating higher burden of coexisting conditions.

### Covariate Balance After Matching

All matches achieved improved covariate balance as indicated by reductions to less than 0.20 SD, and all were sufficiently balanced (eTables 2-9 in Supplement 1). All matches of patients with sepsis had ideal covariate balance (SD < 0.10) (eTables 2-5 in Supplement 1). Most matches of patients with ARF were ideal (SD < 0.10); exceptions were sufficient matches to Black patients in the matches of initial ICU admission (SD = −0.10 for age) and inpatient death (SD = −0.12 for age) (eTable 6 in Supplement 1), and matches to multiracial patients in the match of inpatient death (SD = −0.10 for sex) (eTable 9 in Supplement 1).

### Race and Ethnicity–Specific Differences in LOS

In the fully adjusted primary analysis, Black patients with sepsis (1.26 [95% CI, 0.68-1.84] days; *P* < .001) or ARF (0.97 [95% CI, 0.05-1.89] days; *P* = .03) who were additionally matched on inpatient death had significantly longer LOS relative to White patients. Length of stay was shorter among Asian or Pacific Islander patients with ARF (−0.61 [95% CI, −0.88 to −0.34] days; *P* < .001) and Hispanic patients with sepsis (−0.22 [95% CI, −0.39 to −0.05] days; *P* = .01) or ARF (−0.47 [−0.73 to −0.20] days; *P* = .001). However, no significant differences in LOS were found between other groups with sepsis (Asian or Pacific Islander patients: −0.19 [95% CI, −0.41 to 0.04] days; *P* = .11; multiracial patients: 0.19 [95% CI, −0.05 to 0.44] days; *P* = .12) or ARF (multiracial patients: 0.09 [95% CI, −0.26 to 0.43] days; *P* = .61).

**Figure 2.**
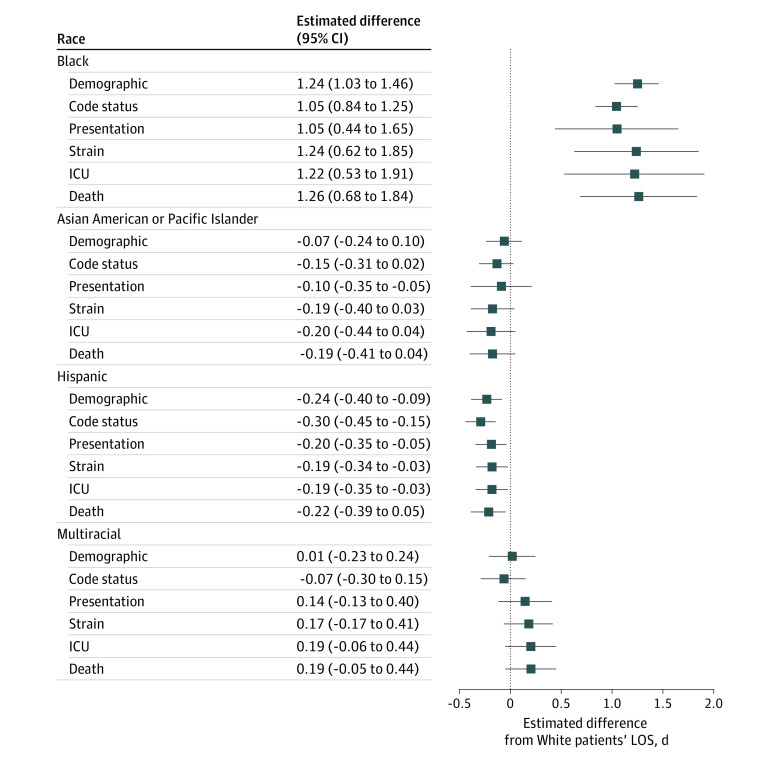
Differences in Hospital Length of Stay (LOS) Among Racial and Ethnic Minority Patients With Sepsis Relative to White Patients Matching variables that were controlled for in the respective matches are detailed in eTable 1 in Supplement 1. ICU indicates intensive care unit.

**Figure 3.**
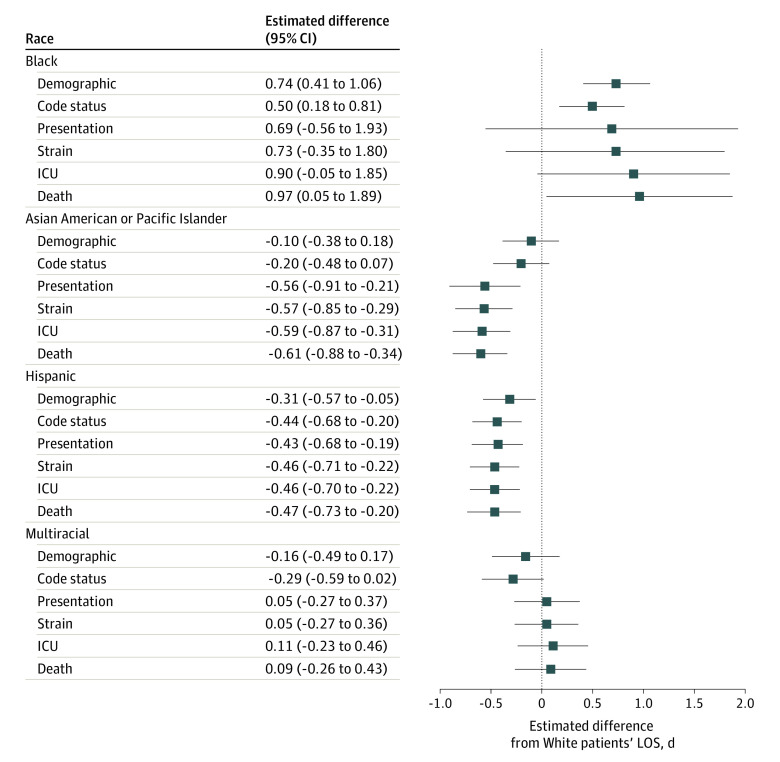
Differences in Hospital Length of Stay (LOS) Among Racial and Ethnic Minority Patients With Acute Respiratory Failure Relative to White Patients Matching variables that were controlled for in the respective matches are detailed in eTable 1 in Supplement 1. ICU indicates intensive care unit.

Matching solely on demographic variables not related to race and ethnicity, Black patients had longer LOS than White patients (sepsis: 1.24 [95% CI, 1.03-1.46] days; *P* < .001; ARF: 0.74 [95% CI, 0.41-1.06] days; *P* < .001) (Table 2, Figure 2, and Figure 3). In contrast, Hispanic patients had shorter LOS than White patients (sepsis: −0.24 [95% CI, −0.40 to −0.09] days; *P* = .002; ARF: −0.31 [95% CI, −0.57 to −0.05] days; *P* = .02). Neither Asian or Pacific Islander patients (sepsis: −0.07 [95% CI, −0.24 to 0.10] days; *P* = .42; ARF: −0.10 [95% CI, −0.38 to 0.18] days; *P* = .47) nor multiracial patients (sepsis: 0.01 [95% CI, −0.23 to 0.24] days; *P* = .95; ARF: −0.16 [95% CI, −0.49 to 0.17] days; *P* = .34) had significantly different LOS.

**Table 2.** Differences in Hospital LOS Between Racial and Ethnic Minority Patients vs White Patients by Nearest-Neighbor Matching

Disease by race or ethnicity	Match type^a^											
	Demographic characteristics		DNR code status		Clinical presentation		Hospital capacity strain		Initial ICU admission		Inpatient death^b^	
	Difference in LOS (95% CI), d	*P* value	Difference in LOS (95% CI), d	*P* value	Difference in LOS (95% CI), d	*P* value	Difference in LOS (95% CI), d	*P* value	Difference in LOS (95% CI), d	*P* value	Difference in LOS (95% CI), d	*P* value
Sepsis												
Asian American and Pacific Islander	−0.07 (−0.24 to 0.10)	.42	−0.15 (−0.31 to 0.02)	.08	−0.10 (−0.39 to 0.20)	.53	−0.19 (−0.40 to 0.03)	.09	−0.20 (−0.44 to 0.04)	.10	−0.19 (−0.41 to 0.04)	.11
Black	1.24 (1.03 to 1.46)	<.001	1.05 (0.84 to 1.25)	<.001	1.05 (0.44 to 1.65)	<.001	1.24 (0.62 to 1.85)	<.001	1.22 (0.53 to 1.91)	<.001	1.26 (0.68 to 1.84)	<.001
Hispanic	−0.24 (−0.40 to −0.09)	.002	−0.30 (−0.45 to −0.15)	<.001	−0.20 (−0.35 to −0.05)	.01	−0.19 (−0.34 to −0.03)	.02	−0.19 (−0.35 to −0.03)	.02	−0.22 (−0.39 to −0.05)	.01
Multiracial	0.01 (−0.23 to 0.24)	.95	−0.07 (−0.30 to 0.15)	.52	0.14 (−0.13 to 0.40)	.31	0.17 (−0.07 to 0.41)	.17	0.19 (−0.06 to 0.44)	.13	0.19 (−0.05 to 0.44)	.12
Acute respiratory failure												
Asian American and Pacific Islander	−0.10 (−0.38 to 0.18)	.47	−0.20 (−0.48 to 0.07)	.15	−0.56 (−0.91 to −0.21)	.001	−0.57 (−0.85 to −0.29)	<.001	−0.59 (−0.87 to −0.31)	<.001	−0.61 (−0.88 to −0.34)	<.001
Black	0.74 (0.41 to 1.06)	<.001	0.50 (0.18 to 0.81)	.002	0.69 (−0.56 to 1.93)	.28	0.73 (−0.35 to 1.80)	.19	0.90 (−0.05 to 1.85)	.06	0.97 (0.05 to 1.89)	.03
Hispanic	−0.31 (−0.57 to −0.05)	.02	−0.44 (−0.68 to −0.20)	<.001	−0.43 (−0.68 to −0.19)	.001	−0.46 (−0.71 to −0.22)	<.001	−0.46 (−0.70 to −0.22)	<.001	−0.47 (−0.73 to −0.20)	.001
Multiracial	−0.16 (−0.49 to 0.17)	.34	−0.29 (−0.59 to 0.02)	.06	0.05 (−0.27 to 0.37)	.75	0.05 (−0.27 to 0.36)	.76	0.11 (−0.23 to 0.46)	.51	0.09 (−0.26 to 0.43)	.61

Abbreviations: DNR, do not resuscitate; ICU, intensive care unit; LOS, length of stay.

^a^ Analyses were stratified by patient race, ethnicity, and disease. Variables controlled for in the respective matches are shown in eTable 1 in Supplement 1. Point estimates reflect differences in mean treatment effects between patients who identified as belonging to the indicated racial or ethnic minority group (intervention group) vs patients who identified as White (reference group). Stepwise addition of matched variables was performed, with demographic characteristics added first, DNR code status added second, clinical presentation added third, hospital capacity strain added fourth, initial ICU admission added fifth, and inpatient death added last.

^b^ Because all matched variables were included in the inpatient death–matched analysis, it was considered the primary analysis, with other match types representing secondary analyses.

Accounting for DNR code status attenuated LOS disparities among Black patients (sepsis: 1.05 [95% CI, 0.84-1.25] days; *P* < .001; ARF: 0.50 [95% CI, 0.18-0.81] days; *P* = .002). Code status accentuated LOS differences among Hispanic patients (sepsis: −0.30 [95% CI, −0.45 to −0.15] days; *P* < .001; ARF: −0.44 [95% CI, −0.68 to −0.20] days; *P* < .001). No notable changes were observed among Asian or Pacific Islander patients (sepsis: −0.15 [95% CI, −0.31 to 0.02] days; *P* = .08; ARF: −0.20 [95% CI, −0.48 to 0.07] days; *P* = .15) or multiracial patients (sepsis: −0.07 [95% CI, −0.30 to 0.15] days *P* = .52; ARF: −0.29 [95% CI, −0.59 to 0.02] days; *P* = .06).

Additionally accounting for clinical- and patient-level characteristics in the clinical presentation–matched analysis revealed no notable changes among patients with sepsis (Asian or Pacific Islander patients: −0.10 [95% CI, −0.39 to 0.20] days; *P* = .53; Black patients: 1.05 [95% CI, 0.44-1.65] days; *P* < .001; Hispanic patients: −0.20 [95% CI, −0.35 to −0.05] days; *P* = .01; multiracial patients: 0.14 [95% CI, −0.13 to 0.40] days; *P* = .31). Among patients with ARF, the LOS difference was no longer significant for Black patients (0.69 [95% CI, −0.56 to 1.93] days; *P* = .28) relative to White patients. In contrast, Asian or Pacific Islander patients with ARF had lower hospital LOS compared with White patients(−0.56 [95% CI, −0.91 to −0.21] days; *P* = .001).

Disparities widened after additionally accounting for hospital-wide capacity strain among Black patients compared with White patients with sepsis (1.24 [95% CI, 0.62-1.85] days; *P* < .001). Group differences remained similar to those found among other matches of patients with sepsis (Asian or Pacific Islander patients: −0.19 [95% CI, −0.40 to 0.03] days; *P* = .09; Hispanic patients: −0.19 [95% CI, −0.34 to −0.03] days; *P* = .02; multiracial patients: 0.17 [95% CI, −0.07 to 0.41] days; *P* = .17) and among all matches of patients with ARF (Asian or Pacific Islander patients: −0.57 [95% CI, −0.85 to −0.29] days; *P* < .001; Black patients: 0.73 [95% CI, −0.35 to 1.80] days; *P* = .19; Hispanic patients: −0.46 [95% CI, −0.71 to −0.22] days; *P* < .001; multiracial patients: 0.05 [95% CI, −0.27 to 0.36] days; *P* = .76).

Further accounting for initial ICU admission revealed no notable changes between matches of patients with sepsis (Asian or Pacific Islander patients: −0.20 [95% CI, −0.44 to 0.04] days; *P* = .10; Black patients: 1.22 [95% CI, 0.53-1.91] days; *P* < .001; Hispanic patients: −0.19 [95% CI, −0.35 to −0.03] days; *P* = .02; multiracial patients: 0.19 [95% CI, −0.06 to 0.44] days; *P* = .13). Similarly, no notable changes were revealed between matches of patients with ARF (Asian or Pacific Islander patients: −0.59 [95% CI, −0.87 to −0.31] days; *P* < .001; Black patients: 0.90 [95% CI, −0.05 to 1.85] days; *P* = .06; Hispanic patients: (−0.46 [95% CI, −0.70 to −0.22] days; *P* < .001; multiracial patients: 0.11 [95% CI, −0.23 to 0.46] days; *P* = .51).

### Secondary Analyses Using Implicit Matching

Across-domain LOS-matched differences were markedly different only among Black and White patients and ranged between 16% and 20% across the matches of clinical presentation, capacity strain, initial ICU admission, and inpatient death. Covariate balance was ideal before and after implicit matches of these groups (eTable 10 in Supplement 1). No significant LOS differences were observed for the implicit matches of clinical presentation and capacity strain (0.05 [95% CI, −0.29 to 0.39] days; *P* = .77), clinical presentation and initial ICU admission (0.13 [95% CI, −0.19 to 0.45] days; *P* = .44), and clinical presentation and inpatient death (0.10 [95% CI, −0.20 to 0.39] days; *P* = .52) (eTable 11 in Supplement 1). The lack of statistical significance among these implicit matches suggests that the additionally matched hospital capacity strain, initial ICU admission, and inpatient death variables incompletely accounted for between-group differences.

### Sensitivity Analyses

Death-placed LOS differences were similar overall to those of the primary analysis. Patients with sepsis had similar magnitude, direction, and statistical significance of LOS differences compared with those of the primary analysis, with the exception of shorter LOS in the match of inpatient death for Asian or Pacific Islander patients (−0.24 [95% CI, −0.45 to −0.03] days; *P* = .02) (eTable 12 and eFigure 1 in Supplement 1). Matches to Black patients with ARF revealed LOS differences that were comparable with those observed in the primary analysis (eTable 12 and eFigure 2 in Supplement 1). However, among matches of Asian or Pacific Islander patients, Hispanic patients, and multiracial patients with ARF, death-placed LOS differences were not significant.

Findings of the SACE analyses were consistent overall with those of the primary analyses, though with notable differences. While median LOS was longer among Black vs White patients with sepsis (0.46 [95% CI, 0.31-0.62] days; *P* < .001) (eTable 12 and eFigure 1 in Supplement 1), differences were of a smaller magnitude. Median LOS was lower for Asian or Pacific Islander patients (−0.26 days [95% CI, −0.45 to −0.07] days; *P* = .008) and Hispanic patients (−0.22 [95% CI, −0.40 to −0.05] days; *P* = .01) with ARF, again with a smaller magnitude than in the primary analysis (eTable 12 and eFigure 2 in Supplement 1). Contrary to the primary analysis, Hispanic patients with sepsis and Black patients with ARF did not have significantly different median LOS from White patients based on SACE.

## Discussion

In this cohort study, we found that hospital LOS was longest for Black patients admitted with sepsis and/or ARF who did not immediately require life support. In contrast, LOS was shorter or similar among Asian or Pacific Islander and Hispanic patients. Despite achieving covariate balance on several commonly implicated patient- and care-related matching variables, race and ethnicity–specific LOS differences remained. Our results revealed that previously identified factors incompletely accounted for LOS disparities and suggest that other underappreciated factors may play important roles.

Although previous studies^5,6,7,8,9^ suggested poorer outcomes for Black patients with sepsis or ARF, the present work advances understanding of these disparities. First, we focused on severely ill patients who did not immediately receive advanced life support; these patients represent an understudied population in the disparities field who may be most susceptible to cognitively biased care delivery. Quantifying the role of ICU care delivery in the development of disparities among these patients was therefore a priority. Second, we used advanced matching methods to address multiple sources of potential confounding; the high quality of matches adds confidence to our results. Third, confidence in our conclusions is supported by consistent signals of between-group differences across multiple methods for analyzing hospital LOS, an outcome that is important to all key stakeholders but analytically challenging in critically ill populations.^48^ Fourth, our study is the first, to our knowledge, to extend the principles of exterior matching to a with-replacement matching method, highlighting the flexibility of these methods for investigations that seek to identify mechanisms that underlie disparities.

In the context of these key design features, we found that LOS disparities in the study population were of a larger magnitude than disparities among non–severely ill populations.^49,50^ This could be due to better control of confounding through advanced matching methods, the criteria we set for cohort entry (especially high acuity and organ dysfunction burden), or other factors. In addition, the magnitude of LOS disparities found in our study is consistent with recently reported LOS disparities among patients with sepsis presenting to hospitals serving racial and ethnic minority patients, who experienced longer median hospital LOS of approximately 1 day.^51^

Notably, adjustment for clinical presentation characteristics did not completely explain LOS disparities between Black and White patients with sepsis. This finding is notable because differences associated with age, sex, chronic comorbidity, acute disease severity, DNR code status, and study hospitals (all variables that were adjusted for in the match of clinical presentation) are surmised to be the source of racial and ethnic disparities.^2,5,13,14,15,16,17^ We also found that capacity strain did not meaningfully account for between-group differences. However, differences were mildly (but not completely) attenuated by DNR code status, suggesting that comprehensively characterizing advanced care planning differences between Black and White patients should be a priority for future studies. Notably, the lower proportion of Black patients with DNR orders relative to other groups is consistent with findings in previous literature.^52,53,54,55,56^ In addition, implicit matching revealed that initial ICU admission did not mitigate disparities. By remaining robust to all matching variables, our findings highlight the fact that other unmeasured mechanisms of disparities need to be identified. Among them, socioeconomic contextualizing factors relevant to hospital presentation and discharge care coordination are likely to have large impacts on the timing and quality of discharge and, therefore, hospital LOS. Future studies might investigate how factors related to socioeconomic disadvantage are associated with hospital LOS among patients with sepsis and ARF.

To our knowledge, this study is one of few to rigorously examine outcomes among severely ill patients who identify as Asian or Pacific Islander or Hispanic. Some studies have suggested poorer outcomes^6,8^ and others improved outcomes^3^ in these populations. Our study found that in contrast to Black patients, Hispanic patients experienced shorter LOS. In addition, LOS was lower among Asian or Pacific Islander patients with ARF. In part, our findings may be explained by the prevalence of baseline characteristics unique to this study population. In our data set, Hispanic patients were more likely to have lower chronic disease burden (eg, COPS2 results). Moreover, more Hispanic patients had private insurance (79.4%), and fewer Asian or Pacific Islander patients had public insurance (1.7% with Medicare and 2.3% with Medicaid), than national averages (48% and 19%, respectively). The rates of participants with private vs public insurance were comparable with the national average among Black and White patient groups (although fewer Black patients with ARF had private insurance).^57^ This finding reinforces our previous hypothesis that socioeconomic health factors that were not accounted for in our matches may be relevant to between-group differences.

Among potential factors that may play a role in LOS disparities, racism is a key mechanism requiring specific attention. The consequences and experience of racism are too complex to be captured in our data. Nevertheless, we identified disparities that appeared to persist despite adjustment for well-validated factors associated with disease severity and outcomes. These disparities likely have origins in patterns of systemic racism that pervade the lived American experience. Whether our results reflect (1) cumulative and lifelong consequences of systemic racism that may be associated with poor outcomes before, during, and after severe illness (consistent with health consequences reported in other studies^58,59^ and related conceptual models^60,61^); (2) evidence of racially disparate health care delivery that would define medical institutional racism; or (3) some combination of these and related factors is unclear based solely on the current analyses. In addition, although we found no evidence for disparities among patients identifying as Asian or Pacific Islander, Hispanic, or multiracial, our findings should not be considered as evidence that health effects of systemic racism do not affect these groups.

### Limitations

This study has several limitations. Unmeasured and residual confounding might further impact LOS differences. However, we adjusted for factors that are most established as explanatory for critical illness–related disparities. Additionally, patient race and ethnicity were determined by self-report but were unknown for some patients who were unable to self-identify. Misclassification bias is a common limitation whenever EHR data are used to assign patient racial or ethnic identity.^62^ While the number of patients with misclassified identity was unquantifiable, clinician assumption of racial or ethnic identity is nevertheless a necessary component of racially biased decision-making; thus, we would expect clinician assignment of identity to be consistent with the consequences of biased care delivery. Furthermore, EHR documentation generally has high specificity compared with patient self-report^63^ but poorer positive predictive value among non-Black minority individuals.^64^ Analyses involving Asian or Pacific Islander, Hispanic, and multiracial patients may therefore be most susceptible to misclassification bias in this study. Moreover, race-related analytic biases related to disease severity scores may have impacted our results; however, we used the LAPS2, a score that performs with less bias than some others.^23^

In addition, we were unable to assess in the present data set whether patients subsequently developed a need for invasive mechanical ventilation or vasopressors after their ED presentation; future studies that focus on specific critical care treatments that occur later in admission are warranted. The effects of poverty are also important factors associated with health outcomes, and our study is limited by only using patient insurance status to infer these characteristics. Although performed across multiple centers, the study sample may not be representative of patients in many settings and, in particular, reflects care delivered in integrated health care systems.

## Conclusions

By matching on important patient-level and care-related factors, this cohort study found that Black patients with sepsis and patients with ARF experienced longer hospital LOS relative to White patients when severely ill but not immediately requiring invasive support. In addition, Hispanic patients experienced lower LOS compared with White patients, and Asian or Pacific Islander patients experienced comparable LOS when presenting with sepsis but shorter LOS when presenting with ARF. Commonly understood factors associated with disparities did not completely account for this study’s results; future work should therefore characterize additional factors associated with these disparities, with emphasis on how patterns of structural racism may play a role.

## Notes

Supplement 1. **eMethods.** Detailed Matching Algorithm and Implicit Matching **eTable 1.** Summary of Matching Variables **eTable 2.** Covariate Balance Between Black and White Patient Groups Before and After Matching in the Sepsis Population **eTable 3.** Covariate Balance Between Asian or Pacific Islander and White Patient Groups Before and After Matching in the Sepsis Population **eTable 4.** Covariate Balance Between Hispanic and White Patient Groups Before and After Matching in the Sepsis Population **eTable 5.** Covariate Balance Between Multiple Race and White Patient Groups Before and After Matching in the Sepsis Population **eTable 6.** Covariate Balance Between Black and White Patient Groups Before and After Matching in the Acute Respiratory Failure Population **eTable 7.** Covariate Balance Between Asian or Pacific Islander and White Patient Groups Before and After Matching in the Acute Respiratory Failure Population **eTable 8.** Covariate Balance Between Hispanic and White Patient Groups Before and After Matching in the Acute Respiratory Failure Population **eTable 9.** Covariate Balance Between Multiple Race and White Patient Groups Before and After Matching in the Acute Respiratory Failure Population **eTable 10.** Covariate Balance Among White Patient Groups Implicitly Matched to Black Patients **eTable 11.** Between-Match Differences Between Nearest-Neighbor White Patients via Exterior Matching **eTable 12.** Estimated Differences in Hospital Length of Stay Using Placement of Death and Survivor Average Causal Effects **eFigure 1.** Sensitivity Analyses for Race-Specific Differences in Hospital Length of Stay in the Sepsis Population **eFigure 2.** Sensitivity Analyses for Race-Specific Differences in Hospital Length of Stay in the Acute Respiratory Failure Population
eReferences

Supplement 2.
Data Sharing Statement
