# Supplementary material for: Racial Disparities in Length of Stay Among Severely Ill Patients Presenting With Sepsis and Acute Respiratory Failure
Source: JAMA Netw Open. 2023 May 8;6(5):e239739. doi: 10.1001/jamanetworkopen.2023.9739 (PMC10167564; doi:10.1001/jamanetworkopen.2023.9739)
Supplement: Supplement 1. — eMethods. Detailed Matching Algorithm and Implicit Matching eTable 1. Summary of Matching Variables eTable 2. Covariate Balance Between Black and White Patient Groups Before and After Matching in the Sepsis Population eTable 3. Covariate Balance Between Asian or Pacific Islander and White Patient Groups Before and After Matching in the Sepsis Population eTable 4. Covariate Balance Between Hispanic and White Patient Groups Before and After Matching in the Sepsis Population eTable 5. Covariate Balance Between Multiple Race and White Patient Groups Before and After Matching in the Sepsis Population eTable 6. Covariate Balance Between Black and White Patient Groups Before and After Matching in the Acute Respiratory Failure Population eTable 7. Covariate Balance Between Asian or Pacific Islander and White Patient Groups Before and After Matching in the Acute Respiratory Failure Population eTable 8. Covariate Balance Between Hispanic and White Patient Groups Before and After Matching in the Acute Respiratory Failure Population eTable 9. Covariate Balance Between Multiple Race and White Patient Groups Before and After Matching in the Acute Respiratory Failure Population eTable 10. Covariate Balance Among White Patient Groups Implicitly Matched to Black Patients eTable 11. Between-Match Differences Between Nearest-Neighbor White Patients via Exterior Matching eTable 12. Estimated Differences in Hospital Length of Stay Using Placement of Death and Survivor Average Causal Effects eFigure 1. Sensitivity Analyses for Race-Specific Differences in Hospital Length of Stay in the Sepsis Population eFigure 2. Sensitivity Analyses for Race-Specific Differences in Hospital Length of Stay in the Acute Respiratory Failure Population eReferences [file jamanetwopen-e239739-s001.pdf]

Supplementary Online Content

Chesley CF, Chowdhury M, Small DS, et al. Racial disparities in length of stay among severely ill patients presenting with sepsis and acute respiratory failure. *JAMA Netw Open*. 2023;6(5):e239739. doi:10.1001/jamanetworkopen.2023.9739

eMethods. Detailed Matching Algorithm and Implicit Matching

eTable 1. Summary of Matching Variables

eTable 2. Covariate Balance Between Black and White Patient Groups Before and After Matching in the Sepsis Population

eTable 3. Covariate Balance Between Asian or Pacific Islander and White Patient Groups Before and After Matching in the Sepsis Population

eTable 4. Covariate Balance Between Hispanic and White Patient Groups Before and After Matching in the Sepsis Population

eTable 5. Covariate Balance Between Multiple Race and White Patient Groups Before and After Matching in the Sepsis Population

eTable 6. Covariate Balance Between Black and White Patient Groups Before and After Matching in the Acute Respiratory Failure Population

eTable 7. Covariate Balance Between Asian or Pacific Islander and White Patient Groups Before and After Matching in the Acute Respiratory Failure Population

eTable 8. Covariate Balance Between Hispanic and White Patient Groups Before and After Matching in the Acute Respiratory Failure Population

eTable 9. Covariate Balance Between Multiple Race and White Patient Groups Before and After Matching in the Acute Respiratory Failure Population

eTable 10. Covariate Balance Among White Patient Groups Implicitly Matched to Black Patients

eTable 11. Between-Match Differences Between Nearest-Neighbor White Patients via Exterior Matching

eTable 12. Estimated Differences in Hospital Length of Stay Using Placement of Death and Survivor Average Causal Effects

eFigure 1. Sensitivity Analyses for Race-Specific Differences in Hospital Length of Stay in the Sepsis Population

eFigure 2. Sensitivity Analyses for Race-Specific Differences in Hospital Length of Stay in the Acute Respiratory Failure Population

eReferences

This supplemental material has been provided by the authors to give readers additional information about their work.

© 2023 Chesley CF et al. *JAMA Network Open*.

eMethods. Detailed Matching Algorithm and Implicit Matching

Demographics match variables included age, gender, and insurance type. The code status match included demographic match variables and additionally the presence of a DNR order. The clinical presentation match included code status match variables and additionally patient COPS2, LAPS2, study hospital, and race- or ethnicity- specific propensity score. Built by logistic regression, propensity scores modeled patient minority race or ethnicity as the outcome variable (using White patient identification as reference), and all matching variables as predicting covariates. Each propensity score was stratified on disease population. Although self-identified race or ethnicity was the primary exposure in all matches, prior work demonstrates that additionally matching on propensity scores reduces bias associated with Mahalanobis distances.[1,2] The strain match included presentation match variables and additionally the disease-specific strain index measured at time of hospital admission. The ICU match included strain match variables and additionally initial admission to either ICU or general ward. The death match included ICU match variables and additionally matched patients on inpatient mortality, with hospice discharges considered deaths. The death match, fully adjusted for all study covariates, was the primary analysis; other matches were considered secondary analyses reflecting contributions of subsets of the match variables to effect estimates.

An important secondary goal was ascertaining the relative importance of presentation characteristics to care-related factors. Another set of secondary analyses, therefore, was to quantify differences between matches for a given minority-nonminority patient group comparison. We quantify between-match differences using an adaptation of exterior matching,[3,4] a tool that compares outcomes among two differently matched control groups sharing an identical exposure group, to nearest-neighbor matching, which matches groups with replacement. We term this method implicit matching, and is a technique that relies on the covariate balance that exists between different match groups that is implicitly maintained via a shared exposed group. In our study, we identified the group of White patients who served as comparators in separate matches for the indicated minority group, and performed an additional nearest-neighbor match of these two groups of White patients. Consistent with exterior matching,[3] the implicit match facilitates a statistical comparison between matched control groups, quantifying how additional adjustment variables contribute to between-match differences. Implicit matching was performed if effect estimates between the presentation and the strain, ICU, or death matches differed by ≥10%.

© 2023 Chesley CF et al. *JAMA Network Open*.

eTable 1. Summary of Matching Variables

Match type	Matching variables
Demographic characteristics	Age, sex, and insurance type
DNR code status	Age, sex, insurance type, and presence of a DNR order
Clinical presentation	Age, sex, insurance type, presence of a DNR order, COPS2, LAPS2, study hospital, and race-specific or ethnicity-specific propensity score
Hospital capacity strain	Age, sex, insurance type, presence of a DNR order, COPS2, LAPS2, study hospital, race-specific or ethnicity-specific propensity score, and disease-specific capacity strain index
Initial ICU admission	Age, sex, insurance type, presence of a DNR order, COPS2, LAPS2, study hospital, race-specific or ethnicity-specific propensity score, disease-specific capacity strain index, and ICU vs ward admission
Inpatient death[a]	Age, sex, insurance type, presence of a DNR order, COPS2, LAPS2, study hospital, race-specific or ethnicity-specific propensity score, disease-specific capacity strain index, ICU vs ward admission, and inpatient death

Abbreviations: COPS2, Comorbidity Point Score, version 2; DNR, do not resuscitate; ICU, intensive care unit; LAPS2, Laboratory Acute Physiology Score, version 2.
[a]Indicates primary analysis, with other match types representing secondary analyses.

© 2023 Chesley CF et al. *JAMA Network Open*.

eTable 2. Covariate Balance Between Black and White Patient Groups Before and After Matching in the Sepsis Population

Definition of abbreviations: ICU= intensive care unit; SD= standardized difference; DNR= do not resuscitate; COPS2= Comorbidity Point Score; LAPS2= Laboratory Acute Physiology Score

Variable	Demographic match		Code status match		Presentation match		Strain match		ICU match		Death match	
	Raw Δ SD	Matched Δ SD	Raw Δ SD	Matched Δ SD	Raw Δ SD	Matched Δ SD	Raw Δ SD	Matched Δ SD	Raw Δ SD	Matched Δ SD	Raw Δ SD	Matched Δ SD
**Age**	-0.50	<0.01	-0.50	<0.01	-0.50	-0.03	-0.50	-0.04	-0.10	0.03	-0.50	-0.06
**Gender**	-0.05	<0.01	-0.05	<0.01	-0.05	0.01	-0.05	0.01	0.08	0.02	-0.05	0.01
**Medicare**	0.24	<0.01	0.24	<0.01	0.24	<0.01	0.24	<0.01	-0.41	0.01	0.24	<0.01
**Medicaid**	0.35	<0.01	0.35	<0.01	0.35	<0.01	0.35	<0.01	0.02	<0.01	0.35	<0.01
**Unknown**	0.23	<0.01	0.23	<0.01	0.23	<0.01	0.23	<0.01	0.18	<0.01	0.23	<0.01
**DNR code status**			0.47	<0.01	0.47	0.02	0.47	0.03	0.18	0.03	0.47	0.05
**COPS2**					0.10	0.01	0.10	0.02	-0.11	-0.04	0.10	0.03
**LAPS2**					0.04	-0.03	0.04	-0.04	0.06	-0.06	0.04	-0.04
**Study hospital 2**					-0.30	<0.01	-0.30	<0.01	-0.13	<0.01	-0.30	<0.01
**Study hospital 3**					-0.13	<0.01	-0.13	<0.01	0.17	<0.01	-0.13	<0.01
**Study hospital 4**					-0.32	<0.01	-0.32	<0.01	-0.40	<0.01	-0.32	<0.01
**Study hospital 5**					-0.15	<0.01	-0.15	<0.01	-0.13	<0.01	-0.15	<0.01
**Study hospital 6**					-0.01	<0.01	-0.01	<0.01	0.06	<0.01	-0.01	<0.01
**Study hospital 7**					-0.03	<0.01	-0.03	<0.01	<0.01	<0.01	-0.03	<0.01
**Study hospital 8**					-0.13	<0.01	-0.13	<0.01	-0.14	<0.01	-0.13	<0.01
**Study hospital 9**					0.37	<0.01	0.37	<0.01	0.16	<0.01	0.37	<0.01
**Study hospital 10**					0.30	<0.01	0.30	<0.01	0.11	<0.01	0.30	<0.01
**Study hospital 11**					-0.38	<0.01	-0.38	<0.01	-0.30	<0.01	-0.38	<0.01
**Study hospital 12**					-0.13	<0.01	-0.13	<0.01	0.03	<0.01	-0.13	<0.01
**Study hospital 13**					-0.06	<0.01	-0.06	<0.01	-0.15	<0.01	-0.06	<0.01
**Study hospital 14**					-0.29	<0.01	-0.29	<0.01	0.16	<0.01	-0.29	<0.01
**Study hospital 15**					0.17	<0.01	0.17	<0.01	-0.15	<0.01	0.17	<0.01
**Study hospital 16**					0.05	<0.01	0.05	<0.01	0.30	<0.01	0.05	<0.01
**Study hospital 17**					0.11	<0.01	0.11	<0.01	0.13	<0.01	0.11	<0.01
**Study hospital 18**					-0.19	<0.01	-0.19	<0.01	-0.16	<0.01	-0.19	<0.01
**Study hospital 19**					-0.28	<0.01	-0.28	<0.01	-0.24	<0.01	-0.28	<0.01
**Study hospital 20**					0.48	<0.01	0.48	<0.01	-0.06	<0.01	0.48	<0.01
**Study hospital 21**					-0.08	<0.01	-0.08	<0.01	0.32	<0.01	-0.08	<0.01
**Study hospital 22**					-0.18	<0.01	-0.18	<0.01	0.05	<0.01	-0.18	<0.01
**Study hospital 23**					-0.06	<0.01	-0.06	<0.01	-0.11	<0.01	-0.06	<0.01
**Study hospital 24**					-0.07	<0.01	-0.07	<0.01	-0.06	<0.01	-0.07	<0.01
**Study hospital 25**					0.38	<0.01	0.38	<0.01	-0.24	<0.01	0.38	<0.01
**Study hospital 26**					0.09	<0.01	0.09	<0.01	0.12	<0.01	0.09	<0.01
**Study hospital 27**					0.14	<0.01	0.14	<0.01	0.15	<0.01	0.14	<0.01
**Propensity score**					1.30	<0.01	1.30	<0.01	0.98	<0.01	1.30	0.01

© 2023 Chesley CF et al. *JAMA Network Open*.

Variable	Demographic match		Code status match		Presentation match		Strain match		ICU match		Death match	
	Raw Δ SD	Matched Δ SD	Raw Δ SD	Matched Δ SD	Raw Δ SD	Matched Δ SD	Raw Δ SD	Matched Δ SD	Raw Δ SD	Matched Δ SD	Raw Δ SD	Matched Δ SD
**Strain index**							-0.07	0.01	-0.18	-0.03	-0.07	0.01
**ICU admission**									0.06	-0.02	0.21	-0.03
**Inpatient mortality**											-0.12	-0.03

© 2023 Chesley CF et al. *JAMA Network Open*.

eTable 3. Covariate Balance Between Asian or Pacific Islander and White Patient Groups Before and After Matching in the Sepsis Population

Definition of abbreviations: ICU= intensive care unit; SD= standardized difference; DNR= do not resuscitate; COPS2= Comorbidity Point Score; LAPS2= Laboratory Acute Physiology Score.

Variable	Demographic match		Code status match		Presentation match		Strain match		ICU match		Death match	
	Raw Δ SD	Matched Δ SD	Raw Δ SD	Matched Δ SD	Raw Δ SD	Matched Δ SD	Raw Δ SD	Matched Δ SD	Raw Δ SD	Matched Δ SD	Raw Δ SD	Matched Δ SD
**Age**	-0.10	<0.01	-0.10	<0.01	-0.10	0.04	-0.10	0.03	-0.10	0.03	-0.10	0.03
**Gender**	0.08	<0.01	0.08	<0.01	0.08	0.01	0.08	0.02	0.08	0.02	0.08	0.02
**Medicare**	-0.41	<0.01	-0.41	<0.01	-0.41	0.01	-0.41	0.01	-0.41	0.01	-0.41	0.01
**Medicaid**	0.02	<0.01	0.02	<0.01	0.02	<0.01	0.02	<0.01	0.02	<0.01	0.02	<0.01
**Unknown**	0.18	<0.01	0.18	<0.01	0.18	<0.01	0.18	<0.01	0.18	<0.01	0.18	<0.01
**DNR codestatus**			0.18	<0.01	0.18	0.02	0.18	0.03	0.18	0.03	0.18	0.04
**COPS2**					-0.11	-0.03	-0.11	-0.04	-0.11	-0.04	-0.11	-0.05
**LAPS2**					0.06	-0.04	0.06	-0.04	0.06	-0.06	0.06	-0.06
**Study hospital 2**					-0.13	<0.01	-0.13	<0.01	-0.13	<0.01	-0.13	<0.01
**Study hospital 3**					0.17	<0.01	0.17	<0.01	0.17	<0.01	0.17	<0.01
**Study hospital 4**					-0.40	<0.01	-0.40	<0.01	-0.40	<0.01	-0.40	<0.01
**Study hospital 5**					-0.13	<0.01	-0.13	<0.01	-0.13	<0.01	-0.13	<0.01
**Study hospital 6**					0.06	<0.01	0.06	<0.01	0.06	<0.01	0.06	<0.01
**Study hospital 7**					<0.01	<0.01	<0.01	<0.01	<0.01	<0.01	<0.01	<0.01
**Study hospital 8**					-0.14	<0.01	-0.14	<0.01	-0.14	<0.01	-0.14	<0.01
**Study hospital 9**					0.16	<0.01	0.16	<0.01	0.16	<0.01	0.16	<0.01
**Study hospital 10**					0.11	<0.01	0.11	<0.01	0.11	<0.01	0.11	<0.01
**Study hospital 11**					-0.30	<0.01	-0.30	<0.01	-0.30	<0.01	-0.30	<0.01
**Study hospital 12**					0.03	<0.01	0.03	<0.01	0.03	<0.01	0.03	<0.01
**Study hospital 13**					-0.15	<0.01	-0.15	<0.01	-0.15	<0.01	-0.15	<0.01
**Study hospital 14**					0.16	<0.01	0.16	<0.01	0.16	<0.01	0.16	<0.01
**Study hospital 15**					-0.15	<0.01	-0.15	<0.01	-0.15	<0.01	-0.15	<0.01
**Study hospital 16**					0.30	<0.01	0.30	<0.01	0.30	<0.01	0.30	<0.01
**Study hospital 17**					0.13	<0.01	0.13	<0.01	0.13	<0.01	0.13	<0.01
**Study hospital 18**					-0.16	<0.01	-0.16	<0.01	-0.16	<0.01	-0.16	<0.01
**Study hospital 19**					-0.24	<0.01	-0.24	<0.01	-0.24	<0.01	-0.24	<0.01
**Study hospital 20**					-0.06	<0.01	-0.06	<0.01	-0.06	<0.01	-0.06	<0.01
**Study hospital 21**					0.32	<0.01	0.32	<0.01	0.32	<0.01	0.32	<0.01
**Study hospital 22**					0.05	<0.01	0.05	<0.01	0.05	<0.01	0.05	<0.01
**Study hospital 23**					-0.11	<0.01	-0.11	<0.01	-0.11	<0.01	-0.11	<0.01
**Study hospital 24**					-0.06	<0.01	-0.06	<0.01	-0.06	<0.01	-0.06	<0.01
**Study hospital 25**					-0.24	<0.01	-0.24	<0.01	-0.24	<0.01	-0.24	<0.01
**Study hospital 26**					0.12	<0.01	0.12	<0.01	0.12	<0.01	0.12	<0.01
**Study hospital 27**					0.15	<0.01	0.15	<0.01	0.15	<0.01	0.15	<0.01
**Propensity score**					0.98	<0.01	0.98	<0.01	0.98	<0.01	0.98	<0.01

© 2023 Chesley CF et al. *JAMA Network Open*.

Variable	Demographic match		Code status match		Presentation match		Strain match		ICU match		Death match	
	Raw Δ SD	Matched Δ SD	Raw Δ SD	Matched Δ SD	Raw Δ SD	Matched Δ SD	Raw Δ SD	Matched Δ SD	Raw Δ SD	Matched Δ SD	Raw Δ SD	Matched Δ SD
**Strain index**							-0.18	-0.03	-0.18	-0.03	-0.18	-0.03
**ICU admission**									0.06	-0.02	0.06	-0.03
**Inpatient mortality**											-0.04	-0.03

© 2023 Chesley CF et al. *JAMA Network Open*.

eTable 4. Covariate Balance Between Hispanic and White Patient Groups Before and After Matching in the Sepsis Population

Definition of abbreviations: ICU= intensive care unit; SD= standardized difference; DNR= do not resuscitate; COPS2= Comorbidity Point Score; LAPS2= Laboratory Acute Physiology Score.

Variable	Demographic match		Code status match		Presentation match		Strain match		ICU match		Death match	
	Raw Δ SD	Matched Δ SD	Raw Δ SD	Matched Δ SD	Raw Δ SD	Matched Δ SD	Raw Δ SD	Matched Δ SD	Raw Δ SD	Matched Δ SD	Raw Δ SD	Matched Δ SD
**Age**	-0.34	<0.01	-0.34	<0.01	-0.34	-0.02	-0.34	-0.02	-0.34	-0.03	-0.34	-0.03
**Gender**	0.05	<0.01	0.05	<0.01	0.05	<0.01	0.05	<0.01	0.05	<0.01	0.05	<0.01
**Medicare**	-0.22	<0.01	-0.22	<0.01	-0.22	<0.01	-0.22	<0.01	-0.22	<0.01	-0.22	<0.01
**Medicaid**	0.13	<0.01	0.13	<0.01	0.13	<0.01	0.13	<0.01	0.13	<0.01	0.13	<0.01
**Unknown**	0.16	<0.01	0.16	<0.01	0.16	<0.01	0.16	<0.01	0.16	<0.01	0.16	<0.01
**DNR codestatus**			0.28	<0.01	0.28	0.02	0.28	0.02	0.28	0.03	0.28	0.03
**COPS2**					-0.13	-0.03	-0.13	-0.04	-0.13	-0.04	-0.13	-0.04
**LAPS2**					-0.02	-0.05	-0.02	-0.06	-0.02	-0.06	-0.02	-0.07
**Study hospital 2**					-0.18	<0.01	-0.18	<0.01	-0.18	<0.01	-0.18	<0.01
**Study hospital 3**					0.03	<0.01	0.03	<0.01	0.03	<0.01	0.03	<0.01
**Study hospital 4**					-0.19	<0.01	-0.19	<0.01	-0.19	<0.01	-0.19	<0.01
**Study hospital 5**					0.15	<0.01	0.15	<0.01	0.15	<0.01	0.15	<0.01
**Study hospital 6**					0.02	<0.01	0.02	<0.01	0.02	<0.01	0.02	<0.01
**Study hospital 7**					0.08	<0.01	0.08	<0.01	0.08	<0.01	0.08	<0.01
**Study hospital 8**					0.06	<0.01	0.06	<0.01	0.06	<0.01	0.06	<0.01
**Study hospital 9**					0.02	<0.01	0.02	<0.01	0.02	<0.01	0.02	<0.01
**Study hospital 10**					0.14	<0.01	0.14	<0.01	0.14	<0.01	0.14	<0.01
**Study hospital 11**					-0.23	<0.01	-0.23	<0.01	-0.23	<0.01	-0.23	<0.01
**Study hospital 12**					<0.01	<0.01	<0.01	<0.01	<0.01	<0.01	<0.01	<0.01
**Study hospital 13**					-0.07	<0.01	-0.07	<0.01	-0.07	<0.01	-0.07	<0.01
**Study hospital 14**					0.07	<0.01	0.07	<0.01	0.07	<0.01	0.07	<0.01
**Study hospital 15**					-0.07	<0.01	-0.07	<0.01	-0.07	<0.01	-0.07	<0.01
**Study hospital 16**					0.04	<0.01	0.04	<0.01	0.04	<0.01	0.04	<0.01
**Study hospital 17**					0.12	<0.01	0.12	<0.01	0.12	<0.01	0.12	<0.01
**Study hospital 18**					-0.16	<0.01	-0.16	<0.01	-0.16	<0.01	-0.16	<0.01
**Study hospital 19**					-0.11	<0.01	-0.11	<0.01	-0.11	<0.01	-0.11	<0.01
**Study hospital 20**					-0.03	<0.01	-0.03	<0.01	-0.03	<0.01	-0.03	<0.01
**Study hospital 21**					0.14	<0.01	0.14	<0.01	0.14	<0.01	0.14	<0.01
**Study hospital 22**					0.22	<0.01	0.22	<0.01	0.22	<0.01	0.22	<0.01
**Study hospital 23**					-0.08	<0.01	-0.08	<0.01	-0.08	<0.01	-0.08	<0.01
**Study hospital 24**					-0.04	<0.01	-0.04	<0.01	-0.04	<0.01	-0.04	<0.01
**Study hospital 25**					-0.15	<0.01	-0.15	<0.01	-0.15	<0.01	-0.15	<0.01
**Study hospital 26**					-0.03	<0.01	-0.03	<0.01	-0.03	<0.01	-0.03	<0.01
**Study hospital 27**					0.08	<0.01	0.08	<0.01	0.08	<0.01	0.08	<0.01
**Propensity score**					0.70	0.01	0.70	0.01	0.70	0.01	0.70	0.01

© 2023 Chesley CF et al. *JAMA Network Open*.

Variable	Demographic match		Code status match		Presentation match		Strain match		ICU match		Death match	
	Raw Δ SD	Matched Δ SD	Raw Δ SD	Matched Δ SD	Raw Δ SD	Matched Δ SD	Raw Δ SD	Matched Δ SD	Raw Δ SD	Matched Δ SD	Raw Δ SD	Matched Δ SD
**Strain index**							-0.10	<0.01	-0.10	<0.01	-0.10	<0.01
**ICU admission**									0.06	-0.01	0.06	-0.02
**Inpatient mortality**											-0.10	-0.03

© 2023 Chesley CF et al. *JAMA Network Open*.

eTable 5. Covariate Balance Between Multiple Race and White Patient Groups Before and After Matching in the Sepsis Population

Definition of abbreviations: ICU= intensive care unit; SD= standardized difference; DNR= do not resuscitate; COPS2= Comorbidity Point Score; LAPS2= Laboratory Acute Physiology Score.

Variable	Demographic match		Code status match		Presentation match		Strain match		ICU match		Death match	
	Raw Δ SD	Matched Δ SD	Raw Δ SD	Matched Δ SD	Raw Δ SD	Matched Δ SD	Raw Δ SD	Matched Δ SD	Raw Δ SD	Matched Δ SD	Raw Δ SD	Matched Δ SD
**Age**	0.06	<0.01	0.06	<0.01	0.06	0.03	0.06	0.04	0.06	0.04	0.06	0.04
**Gender**	-0.07	<0.01	-0.07	<0.01	-0.07	-0.04	-0.07	-0.04	-0.07	-0.05	-0.07	-0.05
**Medicare**	-0.38	<0.01	-0.38	<0.01	-0.38	0.01	-0.38	<0.01	-0.38	<0.01	-0.38	<0.01
**Medicaid**	0.03	<0.01	0.03	<0.01	0.03	-0.01	0.03	-0.01	0.03	-0.01	0.03	-0.01
**Unknown**	-0.14	<0.01	-0.14	<0.01	-0.14	-0.01	-0.14	-0.01	-0.14	-0.01	-0.14	-0.01
**DNR codestatus**			0.05	<0.01	0.05	0.02	0.05	0.02	0.05	0.02	0.05	0.02
**COPS2**					0.14	-0.02	0.14	-0.02	0.14	-0.02	0.14	-0.02
**LAPS2**					0.05	-0.08	0.05	-0.08	0.05	-0.08	0.05	-0.09
**Study hospital 2**					-0.05	<0.01	-0.05	<0.01	-0.05	<0.01	-0.05	<0.01
**Study hospital 3**					0.06	<0.01	0.06	<0.01	0.06	<0.01	0.06	<0.01
**Study hospital 4**					-0.42	-0.02	-0.42	-0.03	-0.42	-0.03	-0.42	-0.03
**Study hospital 5**					-0.02	<0.01	-0.02	<0.01	-0.02	<0.01	-0.02	<0.01
**Study hospital 6**					0.07	<0.01	0.07	<0.01	0.07	<0.01	0.07	<0.01
**Study hospital 7**					0.05	<0.01	0.05	<0.01	0.05	<0.01	0.05	<0.01
**Study hospital 8**					-0.02	<0.01	-0.02	<0.01	-0.02	<0.01	-0.02	<0.01
**Study hospital 9**					0.10	<0.01	0.10	<0.01	0.10	<0.01	0.10	<0.01
**Study hospital 10**					<0.01	<0.01	<0.01	<0.01	<0.01	<0.01	<0.01	<0.01
**Study hospital 11**					-0.12	0.01	-0.12	0.02	-0.12	0.02	-0.12	0.02
**Study hospital 12**					-0.01	<0.01	-0.01	<0.01	-0.01	<0.01	-0.01	<0.01
**Study hospital 13**					-0.01	<0.01	-0.01	<0.01	-0.01	<0.01	-0.01	<0.01
**Study hospital 14**					0.08	<0.01	0.08	<0.01	0.08	<0.01	0.08	<0.01
**Study hospital 15**					-0.04	<0.01	-0.04	<0.01	-0.04	<0.01	-0.04	<0.01
**Study hospital 16**					0.14	<0.01	0.14	<0.01	0.14	<0.01	0.14	<0.01
**Study hospital 17**					0.11	<0.01	0.11	<0.01	0.11	<0.01	0.11	<0.01
**Study hospital 18**					-0.07	<0.01	-0.07	<0.01	-0.07	<0.01	-0.07	<0.01
**Study hospital 19**					-0.09	<0.01	-0.09	<0.01	-0.09	<0.01	-0.09	<0.01
**Study hospital 20**					-0.09	-0.04	-0.09	-0.04	-0.09	-0.04	-0.09	-0.04
**Study hospital 21**					0.14	<0.01	0.14	<0.01	0.14	<0.01	0.14	<0.01
**Study hospital 22**					0.06	<0.01	0.06	<0.01	0.06	<0.01	0.06	<0.01
**Study hospital 23**					-0.11	<0.01	-0.11	<0.01	-0.11	<0.01	-0.11	<0.01
**Study hospital 24**					0.02	<0.01	0.02	<0.01	0.02	<0.01	0.02	<0.01
**Study hospital 25**					-0.12	0.03	-0.12	0.03	-0.12	0.03	-0.12	0.03
**Study hospital 26**					0.09	<0.01	0.09	<0.01	0.09	<0.01	0.09	<0.01
**Study hospital 27**					0.09	<0.01	0.09	<0.01	0.09	<0.01	0.09	<0.01
**Propensity score**					0.61	0.02	0.61	0.02	0.61	0.02	0.61	0.02

© 2023 Chesley CF et al. *JAMA Network Open*.

Variable	Demographic match		Code status match		Presentation match		Strain match		ICU match		Death match	
	Raw Δ SD	Matched Δ SD	Raw Δ SD	Matched Δ SD	Raw Δ SD	Matched Δ SD	Raw Δ SD	Matched Δ SD	Raw Δ SD	Matched Δ SD	Raw Δ SD	Matched Δ SD
**Strain index**							-0.18	-0.04	-0.18	-0.04	-0.18	-0.04
**ICU admission**									0.01	-0.02	0.01	-0.03
**Inpatient mortality**											-0.02	-0.05

© 2023 Chesley CF et al. *JAMA Network Open*.

eTable 6. Covariate Balance Between Black and White Patient Groups Before and After Matching in the Acute Respiratory Failure Population

Definition of abbreviations: ICU= intensive care unit; SD= standardized difference; DNR= do not resuscitate; COPS2= Comorbidity Point Score; LAPS2= Laboratory Acute Physiology Score.

Variable	Demographic match		Code status match		Presentation match		Strain match		ICU match		Death match	
	Raw Δ SD	Matched Δ SD	Raw Δ SD	Matched Δ SD	Raw Δ SD	Matched Δ SD	Raw Δ SD	Matched Δ SD	Raw Δ SD	Matched Δ SD	Raw Δ SD	Matched Δ SD
**Age**	-0.60	<0.01	-0.60	<0.01	-0.60	-0.07	-0.60	-0.08	-0.60	-0.10	-0.60	-0.12
**Gender**	-0.05	<0.01	-0.05	<0.01	-0.05	<0.01	-0.05	<0.01	-0.05	0.01	-0.05	0.01
**Medicare**	0.29	<0.01	0.29	<0.01	0.29	<0.01	0.29	<0.01	0.29	<0.01	0.29	<0.01
**Medicaid**	0.36	<0.01	0.36	<0.01	0.36	-0.01	0.36	-0.01	0.36	-0.01	0.36	-0.01
**Unknown**	0.24	<0.01	0.24	<0.01	0.24	-0.01	0.24	-0.01	0.24	-0.01	0.24	-0.01
**DNR codestatus**			0.54	<0.01	0.54	0.04	0.54	0.05	0.54	0.06	0.54	0.09
**COPS2**					-0.01	0.02	-0.01	0.02	-0.01	0.02	-0.01	0.03
**LAPS2**					-0.11	-0.04	-0.11	-0.04	-0.11	-0.05	-0.11	-0.06
**Study hospital 2**					-0.28	<0.01	-0.28	<0.01	-0.28	<0.01	-0.28	<0.01
**Study hospital 3**					-0.16	<0.01	-0.16	<0.01	-0.16	<0.01	-0.16	<0.01
**Study hospital 4**					-0.36	<0.01	-0.36	<0.01	-0.36	<0.01	-0.36	<0.01
**Study hospital 5**					-0.16	<0.01	-0.16	<0.01	-0.16	<0.01	-0.16	<0.01
**Study hospital 6**					-0.03	<0.01	-0.03	<0.01	-0.03	<0.01	-0.03	<0.01
**Study hospital 7**					-0.05	<0.01	-0.05	<0.01	-0.05	<0.01	-0.05	<0.01
**Study hospital 8**					-0.12	<0.01	-0.12	<0.01	-0.12	<0.01	-0.12	<0.01
**Study hospital 9**					0.36	<0.01	0.36	<0.01	0.36	<0.01	0.36	<0.01
**Study hospital 10**					0.30	<0.01	0.30	<0.01	0.30	<0.01	0.30	<0.01
**Study hospital 11**					-0.41	<0.01	-0.41	<0.01	-0.41	<0.01	-0.41	<0.01
**Study hospital 12**					-0.11	<0.01	-0.11	<0.01	-0.11	<0.01	-0.11	<0.01
**Study hospital 13**					-0.06	<0.01	-0.06	<0.01	-0.06	<0.01	-0.06	<0.01
**Study hospital 14**					-0.31	<0.01	-0.31	<0.01	-0.31	<0.01	-0.31	<0.01
**Study hospital 15**					0.18	<0.01	0.18	<0.01	0.18	<0.01	0.18	<0.01
**Study hospital 16**					0.04	<0.01	0.04	<0.01	0.04	<0.01	0.04	<0.01
**Study hospital 17**					0.10	<0.01	0.10	<0.01	0.10	<0.01	0.10	<0.01
**Study hospital 18**					-0.19	<0.01	-0.19	<0.01	-0.19	<0.01	-0.19	<0.01
**Study hospital 19**					-0.27	<0.01	-0.27	<0.01	-0.27	<0.01	-0.27	<0.01
**Study hospital 20**					0.59	<0.01	0.59	<0.01	0.59	<0.01	0.59	<0.01
**Study hospital 21**					-0.15	<0.01	-0.15	<0.01	-0.15	<0.01	-0.15	<0.01
**Study hospital 22**					-0.16	<0.01	-0.16	<0.01	-0.16	<0.01	-0.16	<0.01
**Study hospital 23**					-0.04	<0.01	-0.04	<0.01	-0.04	<0.01	-0.04	<0.01
**Study hospital 24**					-0.12	<0.01	-0.12	<0.01	-0.12	<0.01	-0.12	<0.01
**Study hospital 25**					0.41	<0.01	0.41	<0.01	0.41	<0.01	0.41	<0.01
**Study hospital 26**					0.06	<0.01	0.06	<0.01	0.06	<0.01	0.06	<0.01
**Study hospital 27**					0.11	<0.01	0.11	<0.01	0.11	<0.01	0.11	<0.01
**Propensity score**					1.48	0.01	1.48	0.01	1.48	0.02	1.48	0.02

© 2023 Chesley CF et al. *JAMA Network Open*.

Variable	Demographic match		Code status match		Presentation match		Strain match		ICU match		Death match	
	Raw Δ SD	Matched Δ SD	Raw Δ SD	Matched Δ SD	Raw Δ SD	Matched Δ SD	Raw Δ SD	Matched Δ SD	Raw Δ SD	Matched Δ SD	Raw Δ SD	Matched Δ SD
**Strain index**							-0.16	-0.01	-0.16	-0.01	-0.16	-0.01
**ICU admission**									0.25	-0.02	0.25	-0.02
**Inpatient mortality**											-0.27	-0.10

© 2023 Chesley CF et al. *JAMA Network Open*.

eTable 7. Covariate Balance Between Asian or Pacific Islander and White Patient Groups Before and After Matching in the Acute Respiratory Failure Population

Definition of abbreviations: ICU= intensive care unit; SD= standardized difference; DNR= do not resuscitate; COPS2= Comorbidity Point Score; LAPS2= Laboratory Acute Physiology Score.

Variable	Demographic match		Code status match		Presentation match		Strain match		ICU match		Death match	
	Raw Δ SD	Matched Δ SD	Raw Δ SD	Matched Δ SD	Raw Δ SD	Matched Δ SD	Raw Δ SD	Matched Δ SD	Raw Δ SD	Matched Δ SD	Raw Δ SD	Matched Δ SD
**Age**	-0.07	<0.01	-0.07	<0.01	-0.07	<0.01	-0.07	<0.01	-0.07	<0.01	-0.07	0.01
**Gender**	0.16	<0.01	0.16	<0.01	0.16	0.03	0.16	0.04	0.16	0.04	0.16	0.05
**Medicare**	-0.47	<0.01	-0.47	<0.01	-0.47	<0.01	-0.47	<0.01	-0.47	<0.01	-0.47	<0.01
**Medicaid**	0.02	<0.01	0.02	<0.01	0.02	-0.01	0.02	-0.01	0.02	-0.01	0.02	-0.01
**Unknown**	0.22	<0.01	0.22	<0.01	0.22	-0.01	0.22	-0.01	0.22	-0.01	0.22	-0.01
**DNR codestatus**			0.17	<0.01	0.17	0.06	0.17	0.05	0.17	0.06	0.17	0.07
**COPS2**					-0.13	-0.02	-0.13	-0.03	-0.13	-0.03	-0.13	-0.04
**LAPS2**					0.11	<0.01	0.11	<0.01	0.11	-0.01	0.11	<0.01
**Study hospital 2**					-0.12	<0.01	-0.12	<0.01	-0.12	<0.01	-0.12	<0.01
**Study hospital 3**					0.16	<0.01	0.16	<0.01	0.16	<0.01	0.16	<0.01
**Study hospital 4**					-0.48	<0.01	-0.48	<0.01	-0.48	<0.01	-0.48	<0.01
**Study hospital 5**					-0.15	<0.01	-0.15	<0.01	-0.15	<0.01	-0.15	<0.01
**Study hospital 6**					0.05	<0.01	0.05	<0.01	0.05	<0.01	0.05	<0.01
**Study hospital 7**					-0.03	<0.01	-0.03	<0.01	-0.03	<0.01	-0.03	<0.01
**Study hospital 8**					-0.10	<0.01	-0.10	<0.01	-0.10	<0.01	-0.10	<0.01
**Study hospital 9**					0.19	<0.01	0.19	<0.01	0.19	<0.01	0.19	<0.01
**Study hospital 10**					0.14	<0.01	0.14	<0.01	0.14	<0.01	0.14	<0.01
**Study hospital 11**					-0.28	<0.01	-0.28	<0.01	-0.28	<0.01	-0.28	<0.01
**Study hospital 12**					0.02	<0.01	0.02	<0.01	0.02	<0.01	0.02	<0.01
**Study hospital 13**					-0.15	<0.01	-0.15	<0.01	-0.15	<0.01	-0.15	<0.01
**Study hospital 14**					0.17	<0.01	0.17	<0.01	0.17	<0.01	0.17	<0.01
**Study hospital 15**					-0.18	<0.01	-0.18	<0.01	-0.18	<0.01	-0.18	<0.01
**Study hospital 16**					0.28	<0.01	0.28	<0.01	0.28	<0.01	0.28	<0.01
**Study hospital 17**					0.14	<0.01	0.14	<0.01	0.14	<0.01	0.14	<0.01
**Study hospital 18**					-0.17	<0.01	-0.17	<0.01	-0.17	<0.01	-0.17	<0.01
**Study hospital 19**					-0.23	<0.01	-0.23	<0.01	-0.23	<0.01	-0.23	<0.01
**Study hospital 20**					-0.06	<0.01	-0.06	<0.01	-0.06	<0.01	-0.06	<0.01
**Study hospital 21**					0.32	<0.01	0.32	<0.01	0.32	<0.01	0.32	<0.01
**Study hospital 22**					0.07	<0.01	0.07	<0.01	0.07	<0.01	0.07	<0.01
**Study hospital 23**					-0.10	<0.01	-0.10	<0.01	-0.10	<0.01	-0.10	<0.01
**Study hospital 24**					-0.20	<0.01	-0.20	<0.01	-0.20	<0.01	-0.20	<0.01
**Study hospital 25**					0.14	<0.01	0.14	<0.01	0.14	<0.01	0.14	<0.01
**Study hospital 26**					0.16	<0.01	0.16	<0.01	0.16	<0.01	0.16	<0.01
**Study hospital 27**					-0.08	<0.01	-0.08	<0.01	-0.08	<0.01	-0.08	<0.01

© 2023 Chesley CF et al. *JAMA Network Open*.

Variable	Demographic match		Code status match		Presentation match		Strain match		ICU match		Death match	
	Raw Δ SD	Matched Δ SD	Raw Δ SD	Matched Δ SD	Raw Δ SD	Matched Δ SD	Raw Δ SD	Matched Δ SD	Raw Δ SD	Matched Δ SD	Raw Δ SD	Matched Δ SD
**Propensity score**					1.02	<0.01	1.02	<0.01	1.02	<0.01	1.02	<0.01
**Strain index**							-0.30	-0.01	-0.30	-0.01	-0.30	-0.02
**ICU admission**									0.16	0.01	0.16	<0.01
**Inpatient mortality**											-0.03	0.01

© 2023 Chesley CF et al. *JAMA Network Open*.

eTable 8. Covariate Balance Between Hispanic and White Patient Groups Before and After Matching in the Acute Respiratory Failure Population

Definition of abbreviations: ICU= intensive care unit; SD= standardized difference; DNR= do not resuscitate; COPS2= Comorbidity Point Score; LAPS2= Laboratory Acute Physiology Score.

Variable	Demographic match		Code status match		Presentation match		Strain match		ICU match		Death match	
	Raw Δ SD	Matched Δ SD	Raw Δ SD	Matched Δ SD	Raw Δ SD	Matched Δ SD	Raw Δ SD	Matched Δ SD	Raw Δ SD	Matched Δ SD	Raw Δ SD	Matched Δ SD
**Age**	-0.34	<0.01	-0.34	<0.01	-0.34	-0.04	-0.34	-0.04	-0.34	-0.05	-0.34	-0.05
**Gender**	0.08	<0.01	0.08	<0.01	0.08	<0.01	0.08	<0.01	0.08	<0.01	0.08	0.01
**Medicare**	-0.26	<0.01	-0.26	<0.01	-0.26	<0.01	-0.26	<0.01	-0.26	<0.01	-0.26	<0.01
**Medicaid**	0.17	<0.01	0.17	<0.01	0.17	<0.01	0.17	<0.01	0.17	<0.01	0.17	<0.01
**Unknown**	0.16	<0.01	0.16	<0.01	0.16	<0.01	0.16	<0.01	0.16	-0.01	0.16	-0.01
**DNR codestatus**			0.25	<0.01	0.25	0.03	0.25	0.04	0.25	0.05	0.25	0.06
**COPS2**					-0.12	-0.03	-0.12	-0.03	-0.12	-0.03	-0.12	-0.03
**LAPS2**					-0.02	-0.07	-0.02	-0.08	-0.02	-0.09	-0.02	-0.09
**Study hospital 2**					-0.16	<0.01	-0.16	<0.01	-0.16	<0.01	-0.16	<0.01
**Study hospital 3**					0.06	<0.01	0.06	<0.01	0.06	<0.01	0.06	<0.01
**Study hospital 4**					-0.20	<0.01	-0.20	<0.01	-0.20	<0.01	-0.20	<0.01
**Study hospital 5**					0.12	<0.01	0.12	<0.01	0.12	<0.01	0.12	<0.01
**Study hospital 6**					0.05	<0.01	0.05	<0.01	0.05	<0.01	0.05	<0.01
**Study hospital 7**					0.07	<0.01	0.07	<0.01	0.07	<0.01	0.07	<0.01
**Study hospital 8**					0.06	<0.01	0.06	<0.01	0.06	<0.01	0.06	<0.01
**Study hospital 9**					0.02	<0.01	0.02	<0.01	0.02	<0.01	0.02	<0.01
**Study hospital 10**					0.14	<0.01	0.14	<0.01	0.14	<0.01	0.14	<0.01
**Study hospital 11**					-0.23	<0.01	-0.23	<0.01	-0.23	<0.01	-0.23	<0.01
**Study hospital 12**					<0.01	<0.01	<0.01	<0.01	<0.01	<0.01	<0.01	<0.01
**Study hospital 13**					-0.09	<0.01	-0.09	<0.01	-0.09	<0.01	-0.09	<0.01
**Study hospital 14**					0.09	<0.01	0.09	<0.01	0.09	<0.01	0.09	<0.01
**Study hospital 15**					-0.06	<0.01	-0.06	<0.01	-0.06	<0.01	-0.06	<0.01
**Study hospital 16**					0.06	<0.01	0.06	<0.01	0.06	<0.01	0.06	<0.01
**Study hospital 17**					0.13	<0.01	0.13	<0.01	0.13	<0.01	0.13	<0.01
**Study hospital 18**					-0.14	<0.01	-0.14	<0.01	-0.14	<0.01	-0.14	<0.01
**Study hospital 19**					-0.12	<0.01	-0.12	<0.01	-0.12	<0.01	-0.12	<0.01
**Study hospital 20**					-0.01	<0.01	-0.01	<0.01	-0.01	<0.01	-0.01	<0.01
**Study hospital 21**					0.09	<0.01	0.09	<0.01	0.09	<0.01	0.09	<0.01
**Study hospital 22**					0.23	<0.01	0.23	<0.01	0.23	<0.01	0.23	<0.01
**Study hospital 23**					-0.08	<0.01	-0.08	<0.01	-0.08	<0.01	-0.08	<0.01
**Study hospital 24**					-0.07	<0.01	-0.07	<0.01	-0.07	<0.01	-0.07	<0.01
**Study hospital 25**					-0.12	<0.01	-0.12	<0.01	-0.12	<0.01	-0.12	<0.01
**Study hospital 26**					-0.03	<0.01	-0.03	<0.01	-0.03	<0.01	-0.03	<0.01
**Study hospital 27**					0.10	<0.01	0.10	<0.01	0.10	<0.01	0.10	<0.01
**Propensity score**					0.68	0.01	0.68	0.01	0.68	0.01	0.68	0.02

© 2023 Chesley CF et al. *JAMA Network Open*.

Variable	Demographic match		Code status match		Presentation match		Strain match		ICU match		Death match	
	**Raw Δ SD**	**Matched Δ SD**	**Raw Δ SD**	**Matched Δ SD**	**Raw Δ SD**	**Matched Δ SD**	**Raw Δ SD**	**Matched Δ SD**	**Raw Δ SD**	**Matched Δ SD**	**Raw Δ SD**	**Matched Δ SD**
**Strain index**							-0.15	-0.01	-0.15	-0.01	-0.15	-0.01
**ICU admission**									0.12	-0.02	0.12	-0.03
**Inpatient mortality**											-0.11	-0.07

© 2023 Chesley CF et al. *JAMA Network Open*.

eTable 9. Covariate Balance Between Multiple Race and White Patient Groups Before and After Matching in the Acute Respiratory Failure Population

Definition of abbreviations: ICU= intensive care unit; SD= standardized difference; DNR= do not resuscitate; COPS2= Comorbidity Point Score; LAPS2= Laboratory Acute Physiology Score.

Variable	Demographic match		Code status match		Presentation match		Strain match		ICU match		Death match	
	Raw Δ SD	Matched Δ SD	Raw Δ SD	Matched Δ SD	Raw Δ SD	Matched Δ SD	Raw Δ SD	Matched Δ SD	Raw Δ SD	Matched Δ SD	Raw Δ SD	Matched Δ SD
**Age**	0.01	<0.01	0.01	<0.01	0.01	0.03	0.01	0.02	0.01	0.03	0.01	0.03
**Gender**	-0.09	<0.01	-0.09	<0.01	-0.09	-0.10	-0.09	-0.10	-0.09	-0.10	-0.09	-0.10
**Medicare**	-0.44	<0.01	-0.44	<0.01	-0.43	0.01	-0.43	<0.01	-0.43	<0.01	-0.43	<0.01
**Medicaid**	0.06	<0.01	0.06	<0.01	0.06	<0.01	0.06	<0.01	0.06	<0.01	0.06	<0.01
**Unknown**	-0.18	<0.01	-0.18	<0.01	-0.18	-0.03	-0.18	-0.03	-0.18	-0.04	-0.18	-0.04
**DNR codestatus**			0.09	<0.01	0.09	-0.03	0.09	-0.02	0.09	-0.02	0.09	-0.01
**COPS2**					0.17	-0.05	0.17	-0.05	0.17	-0.04	0.17	-0.04
**LAPS2**					0.07	-0.06	0.07	-0.07	0.07	-0.08	0.07	-0.08
**Study hospital 2**					-0.05	<0.01	-0.05	<0.01	-0.05	<0.01	-0.05	<0.01
**Study hospital 3**					0.03	<0.01	0.03	<0.01	0.03	<0.01	0.03	<0.01
**Study hospital 4**					-0.49	-0.03	-0.49	-0.04	-0.49	-0.04	-0.49	-0.04
**Study hospital 5**					-0.03	<0.01	-0.03	<0.01	-0.03	<0.01	-0.03	<0.01
**Study hospital 6**					0.06	<0.01	0.06	<0.01	0.06	<0.01	0.06	<0.01
**Study hospital 7**					0.06	<0.01	0.06	<0.01	0.06	<0.01	0.06	<0.01
**Study hospital 8**					<0.01	<0.01	<0.01	<0.01	<0.01	<0.01	<0.01	<0.01
**Study hospital 9**					0.15	<0.01	0.15	<0.01	0.15	<0.01	0.15	<0.01
**Study hospital 10**					0.05	<0.01	0.05	<0.01	0.05	<0.01	0.05	<0.01
**Study hospital 11**					-0.10	0.02	-0.10	0.03	-0.10	0.03	-0.10	0.03
**Study hospital 12**					0.01	<0.01	0.01	<0.01	0.01	<0.01	0.01	<0.01
**Study hospital 13**					-0.03	<0.01	-0.03	<0.01	-0.03	<0.01	-0.03	<0.01
**Study hospital 14**					0.10	0.01	0.10	0.01	0.10	0.01	0.10	0.01
**Study hospital 15**					-0.06	<0.01	-0.06	<0.01	-0.06	<0.01	-0.06	<0.01
**Study hospital 16**					0.13	<0.01	0.13	<0.01	0.13	<0.01	0.13	<0.01
**Study hospital 17**					0.09	<0.01	0.09	<0.01	0.09	<0.01	0.09	<0.01
**Study hospital 18**					-0.09	<0.01	-0.09	<0.01	-0.09	<0.01	-0.09	<0.01
**Study hospital 19**					-0.08	<0.01	-0.08	<0.01	-0.08	<0.01	-0.08	<0.01
**Study hospital 20**					-0.06	<0.01	-0.06	<0.01	-0.06	<0.01	-0.06	<0.01
**Study hospital 21**					0.09	<0.01	0.09	<0.01	0.09	<0.01	0.09	<0.01
**Study hospital 22**					0.11	<0.01	0.11	<0.01	0.11	<0.01	0.11	<0.01
**Study hospital 23**												
**Study hospital 24**					0.01	<0.01	0.01	<0.01	0.01	<0.01	0.01	<0.01
**Study hospital 25**					-0.10	0.01	-0.10	0.01	-0.10	0.01	-0.10	0.01
**Study hospital 26**					0.11	<0.01	0.11	<0.01	0.11	<0.01	0.11	<0.01
**Study hospital 27**					0.10	<0.01	0.10	<0.01	0.10	<0.01	0.10	<0.01

© 2023 Chesley CF et al. *JAMA Network Open*.

Variable	Demographic match		Code status match		Presentation match		Strain match		ICU match		Death match	
	Raw Δ SD	Matched Δ SD	Raw Δ SD	Matched Δ SD	Raw Δ SD	Matched Δ SD	Raw Δ SD	Matched Δ SD	Raw Δ SD	Matched Δ SD	Raw Δ SD	Matched Δ SD
**Propensity score**					0.66	0.03	0.66	0.03	0.66	0.04	0.66	0.04
**Strain index**							-0.28	-0.07	-0.28	-0.06	-0.28	-0.06
**ICU admission**									0.05	-0.03	0.05	-0.04
**Inpatient mortality**											-0.04	-0.08

© 2023 Chesley CF et al. *JAMA Network Open*.

eTable 10. Covariate Balance Among White Patient Groups Implicitly Matched to Black Patients

Definition of abbreviations: ICU= intensive care unit; SD= standardized difference; DNR= do not resuscitate; COPS2= Comorbidity Point Score; LAPS2= Laboratory Acute Physiology Score.

Variable	Presentation vs strain matches		Presentation v ICU matches		Presentation v death matches	
	Raw Δ SD	Matched Δ SD	Raw Δ SD	Matched Δ SD	Raw Δ SD	Matched Δ SD
**Age**	0.02	0.01	0.04	0.01	0.07	0.01
**Gender**	-0.01	<0.001	<0.001	0.01	<0.001	<0.001
**Insurance:**						
**Medicare**	<0.001	<0.001	<0.001	<0.001	0.01	<0.001
**Medicaid**	-0.01	<0.001	<0.001	<0.001	<0.001	<0.001
**Unknown**	-0.01	<0.001	0.01	<0.001	<0.001	<0.001
**DNR code status**	-0.02	<0.001	-0.02	<0.001	-0.02	<0.001
**COPS2**	-0.02	<0.001	<0.001	<0.001	-0.02	-0.01
**LAPS2**	-0.02	<0.001	-0.02	-0.01	-0.02	-0.01
**Study hospital 2**	<0.001	<0.001	-0.01	<0.001	-0.01	<0.001
**Study hospital 3**	-0.01	<0.001	-0.01	<0.001	<0.001	<0.001
**Study hospital 4**	<0.001	<0.001	0.01	<0.001	0.01	<0.001
**Study hospital 5**	<0.001	<0.001	-0.01	<0.001	<0.001	<0.001
**Study hospital 6**	-0.01	<0.001	-0.02	<0.001	-0.02	<0.001
**Study hospital 7**	-0.02	<0.001	-0.02	<0.001	-0.01	<0.001
**Study hospital 8**	<0.001	<0.001	<0.001	<0.001	<0.001	<0.001
**Study hospital 9**	<0.001	<0.001	<0.001	<0.001	<0.001	<0.001
**Study hospital 10**	0.04	<0.001	0.03	<0.001	0.03	<0.001
**Study hospital 11**	0.01	<0.001	0.01	<0.001	0.01	<0.001
**Study hospital 12**	0.01	<0.001	0.01	<0.001	<0.001	<0.001
**Study hospital 13**	0.01	<0.001	0.01	<0.001	<0.001	<0.001
**Study hospital 14**	-0.02	<0.001	-0.01	<0.001	<0.001	<0.001
**Study hospital 15**	0.01	<0.001	0.02	<0.001	0.03	<0.001
**Study hospital 16**	0.01	<0.001	<0.001	<0.001	<0.001	<0.001
**Study hospital 17**	-0.01	<0.001	<0.001	<0.001	-0.01	<0.001
**Study hospital 18**	0.01	<0.001	0.01	<0.001	0.01	<0.001
**Study hospital 19**	<0.001	<0.001	-0.01	<0.001	<0.001	<0.001
**Study hospital 20**	0.01	0.01	-0.02	<0.001	-0.01	<0.001
**Study hospital 21**	<0.001	<0.001	0.01	<0.001	0.01	<0.001
**Study hospital 22**	-0.01	<0.001	-0.01	<0.001	-0.01	<0.001
**Study hospital 23**	0.02	<0.001	0.02	<0.001	0.01	<0.001
**Study hospital 24**	-0.01	<0.001	-0.01	<0.001	-0.02	<0.001
**Study hospital 25**	-0.01	<0.001	-0.01	<0.001	-0.02	<0.001
**Study hospital 26**	0.03	<0.001	0.01	<0.001	0.02	<0.001
**Study hospital 27**	<0.001	<0.001	0.01	<0.001	<0.001	<0.001
**Strain index**	0.07	0.03	0.06	0.02	0.06	0.02
**Propensity score**	0.01	<0.001	0.01	<0.001	<0.001	<0.001
**ICU admission**			0.03	<0.001	0.01	<0.001
**Inpatient mortality**					<0.001	<0.001

© 2023 Chesley CF et al. *JAMA Network Open*.

eTable 11. Between-Match Differences Between Nearest-Neighbor White Patients via Exterior Matching

The groups of White patients who represented the nearest-neighbor matches to Black patients were identified and compared. The estimated differences in length of stay represent average treatment effects that were obtained by nearest neighbor matching between the groups of White patients who were the nearest neighbors of Black patients in the indicated match group.

**Match comparison**	**Δ LOS (days), 95% CI**	p-value
Strain (n=3639) vs Presentation (n=3601)	0.05 (-0.29, 0.39)	0.77
ICU (n=4254) vs Presentation (n=4211)	0.13 (-0.19, 0.45)	0.44
Death (n=4611) vs Presentation (n=4627)	0.10 (-0.20, 0.39)	0.52

© 2023 Chesley CF et al. *JAMA Network Open*.

eTable 12. Estimated Differences in Hospital Length of Stay Using Placement of Death and Survivor Average Causal Effects

Adjusted differences were determined based on nearest-neighbor matching to determine between-group differences in death-placed length of stay (LOS), or were between group differences in median LOS determined by the survivor average causal effect (SACE) method. Similar to our primary analysis, all matching analyses were 2:1 comparisons of patients who identified as minorities to patients who identified as White. Deaths placed at the 95%ile were specific to patient race, ethnicity, and diagnosis. For patients with sepsis who died, LOS was placed at 14.3 days for White patients; 19 days for Black patients; 14.8 days for Asian American or Pacific Islander patients; 13.9 days for Hispanic patients; or 13.7 days for Multiracial patients. For patients with ARF who died, LOS was placed at 15.4 days for White patients; 18.6 days for Black patients; 17.5 days for Asian American or Pacific Islander patients; 16.7 days for Hispanic patients; and 15.1 days for Multiracial patients. Detailed explanations of matching variables are summarized in Online Resource 1. Adjusted difference in length of stay by the SACE method represents adjusted difference in median hospital length of stay and was determined using quantile regression with probability weighting for survival given patient identification to the indicated minority racial or ethnic group. All analyses are stratified by diagnosis. Definition of abbreviations: SACE= survivor average causal effect; ICU= intensive care unit; LOS= length of stay; CI= confidence interval; AAPI= Asian American or Pacific Islander; ARF= acute respiratory failure.

	Race or ethnicity	Demographic match		Code Status match		Presentation Match		Strain match		ICU Match		Death Match		SACE	
		Δ LOS (days), 95% CI	p-value	Δ LOS (days), 95% CI	p-value	Δ LOS (days), 95% CI	p-value	Δ LOS (days), 95% CI	p-value	Δ LOS (days), 95% CI	p-value	Δ LOS (days), 95% CI	p-value	Δ LOS (days), 95% CI	p-value
Sepsis	Black	1.50 (1.29, 1.72)	<0.001	1.64 (1.42, 1.85)	0.01	1.61 (1.03, 2.19)	<0.001	1.75 (1.14, 2.36)	<0.001	1.70 (1.10, 2.29)	<0.001	1.68 (1.15, 2.22)	<0.001	0.46 (0.31, 0.62)	<0.001
	AAPI	-0.13 (-0.31, 0.05)	0.15	-0.09 (-0.27, 0.09)	0.34	-0.16 (-0.41, 0.09)	0.21	-0.19 (-0.43, 0.05)	0.12	-0.22 (-0.47, 0.03)	0.09	-0.24 (-0.45, -0.03)	0.02	0.01 (-0.10, 0.12)	0.91
	Hispanic	-0.53 (-0.69, -0.37)	<0.001	-0.40 (-0.55, -0.24)	<0.001	-0.32 (-0.49, -0.15)	<0.001	-0.31 (-0.48, -0.14)	<0.001	-0.29 (-0.47, -0.12)	<0.001	-0.27 (-0.41, -0.13)	<0.001	-0.05 (-0.16, 0.05)	0.31
	Multiracial	-0.22 (-0.45, 0.01)	0.06	-0.19 (-0.44, 0.05)	0.13	-0.08 (-0.35, 0.19)	0.58	-0.06 (-0.31, 0.19)	0.62	-0.04 (-0.30, 0.22)	0.75	0.10 (-0.14, 0.34)	0.41	0.14 (-0.19, 0.48)	0.40
ARF	Black	0.32 (0.06, 0.59)	0.02	0.58 (0.31, 0.86)	<0.001	0.68 (-0.56, 1.92)	0.28	0.73 (-0.34, 1.80)	0.18	0.90 (-0.05, 1.85)	0.06	0.95 (0.05, 1.84)	0.04	0.13 (-0.11, 0.38)	0.29
	AAPI	0.19 (-0.14, 0.53)	0.25	0.37 (0.03, 0.71)	0.03	0.32 (-0.27, 0.92)	0.29	0.35 (-0.26, 0.96)	0.26	0.35 (-0.26, 0.97)	0.26	0.01 (-0.51, 0.53)	0.96	-0.26 (-0.45, -0.07)	0.008
	Hispanic	-0.24 (-0.51, 0.04)	0.10	-0.12 (-0.40, 0.16)	0.41	-0.20 (-0.51, 0.10)	0.20	-0.18 (-0.48, 0.12)	0.24	-0.17 (-0.48, 0.13)	0.26	-0.15 (-0.38, 0.08)	0.20	-0.22 (-0.40, -0.05)	0.01
	Multiracial	-0.35 (-0.71, 0.01)	0.05	-0.26 (-0.64, 0.12)	0.18	-0.14 (-0.49, 0.22)	0.45	-0.15 (-0.50, 0.20)	0.40	-0.14 (-0.51, 0.24)	0.47	-0.10 (-0.43, 0.23)	0.54	0.12 (-0.10, 0.34)	0.28

© 2023 Chesley CF et al. *JAMA Network Open*.

eFigure 1. Sensitivity Analyses for Race-Specific Differences in Hospital Length of Stay in the Sepsis Population

Analyses are stratified by diagnosis. For all analyses, patients who identify as White were the reference group. The outcome for all matched analyses was the difference in the composite outcome of hospital length of stay by the placement of death method between patients who identified as from minority groups and as White. For these analyses, patients who died had a hospital length of stay equivalent to the 95%ile of the race- or ethnicity-specific distribution. Variables controlled for in the respective matches are explained in Online Resource 1. For the survivor average causal effect analysis, the outcome variable was the difference in the median hospital length of stay based on quantile regression adjusted for patient age, gender, insurance type, code status in the emergency department, Laboratory Acute Physiology Score, Comorbidity Point Score, disease-specific strain index, intensive care unit admission, and hospital facility. Definition of abbreviations: LOS= length of stay; AAPI= Asian American or Pacific Islander; ICU= intensive care unit.

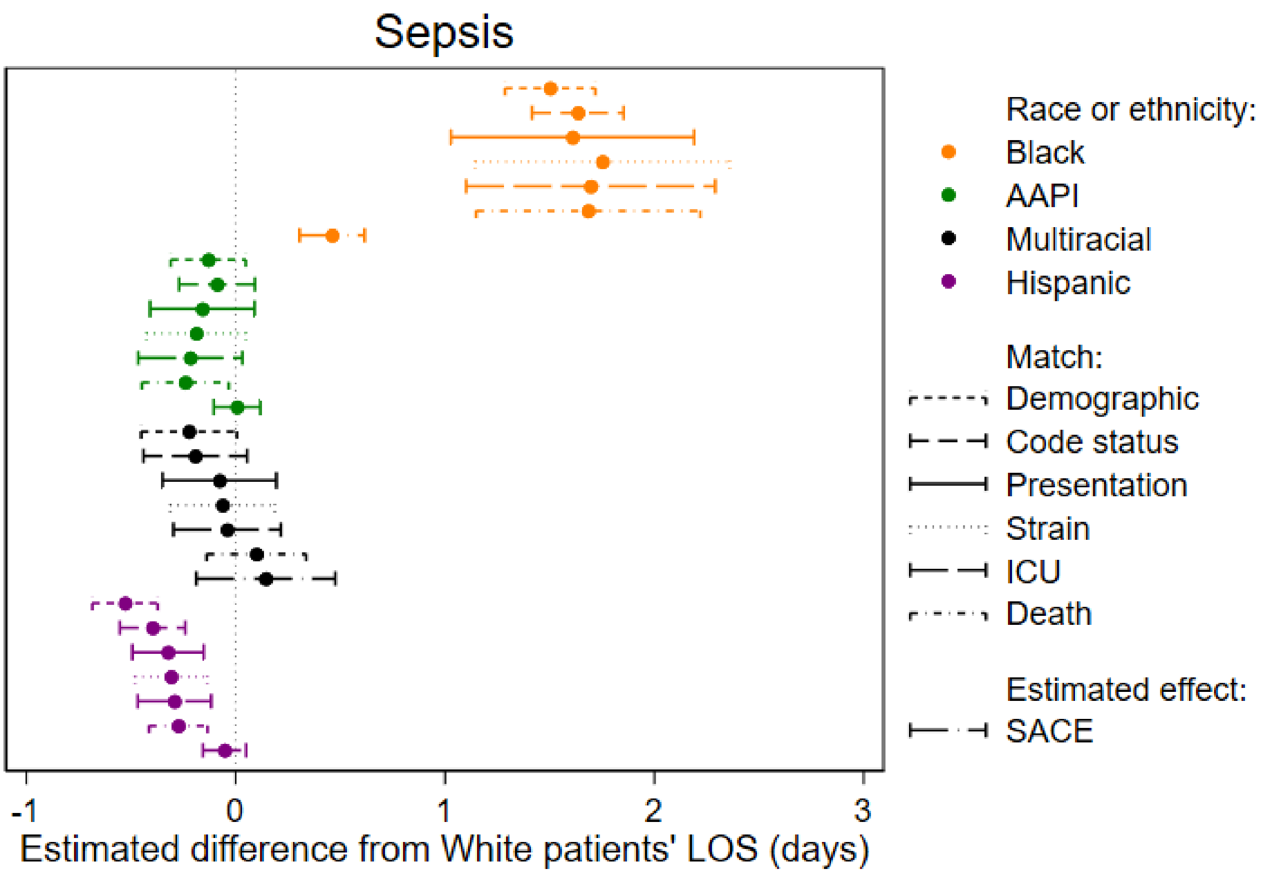

© 2023 Chesley CF et al. *JAMA Network Open*.

eFigure 2. Sensitivity Analyses for Race-Specific Differences in Hospital Length of Stay in the Acute Respiratory Failure Population

Analyses are stratified by diagnosis. For all analyses, patients who identify as White were the reference group. The outcome for all matched analyses was the difference in the composite outcome of hospital length of stay by the placement of death method between patients who identified as from minority groups and as White. For these analyses, patients who died had a hospital length of stay equivalent to the 95%ile of the race- or ethnicity-specific distribution. Variables controlled for in the respective matches are explained in Online Resource 1. For the survivor average causal effect analysis, the outcome variable was the difference in the median hospital length of stay based on quantile regression adjusted for patient age, gender, insurance type, code status in the emergency department, Laboratory Acute Physiology Score, Comorbidity Point Score, disease-specific strain index, intensive care unit admission, and hospital facility. Definition of abbreviations: LOS= length of stay; AAPI= Asian American or Pacific Islander; ICU= intensive care unit.

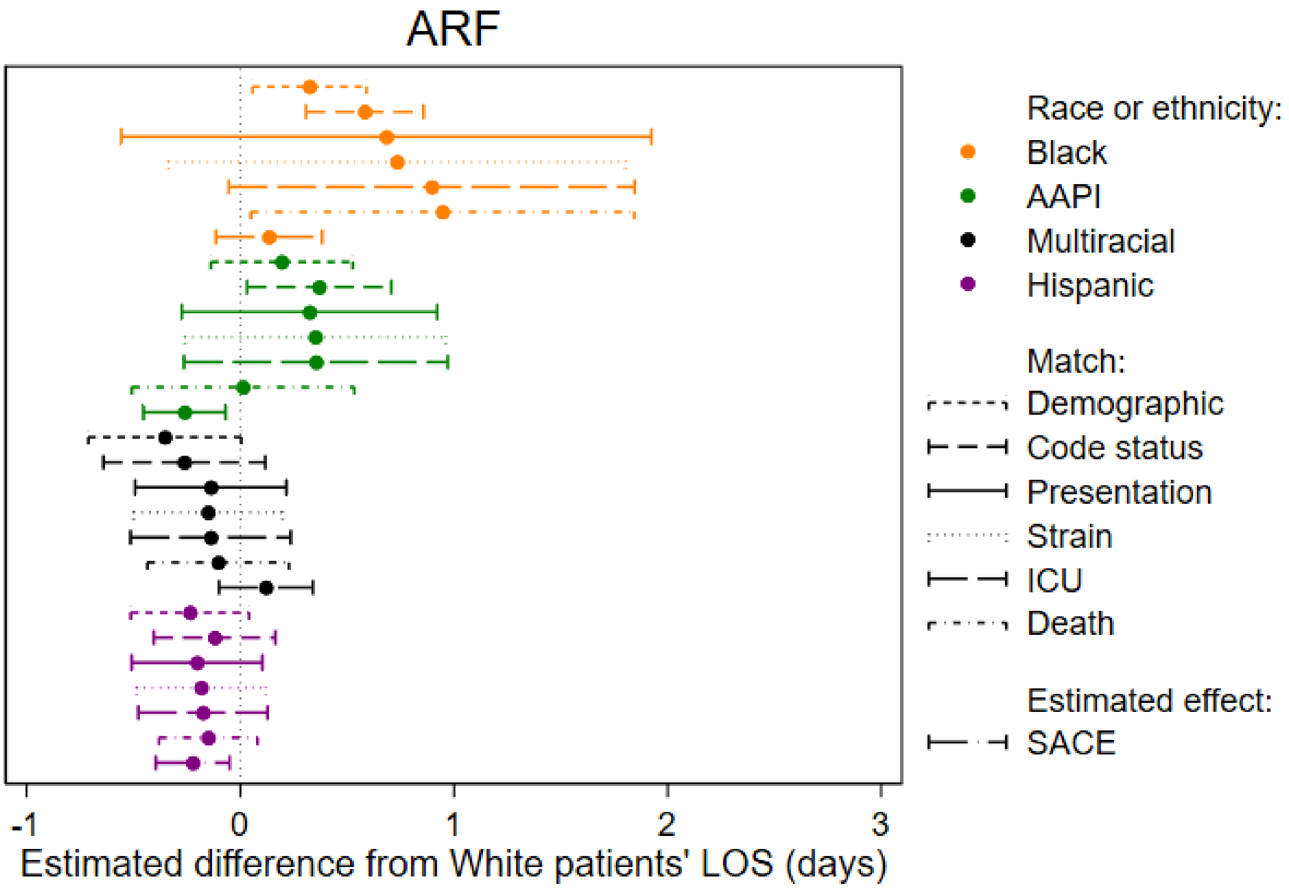

© 2023 Chesley CF et al. *JAMA Network Open*.

eReferences

1. Rosenbaum P, Rubin D. The central role of the propensity score in observational studies for causal effects. *Biometrika*. 1983;70(1):41-55. doi:10.1093/biomet/70.1.41
2. Joffe MM, Rosenbaum PR. Invited Commentary: Propensity Scores. *American Journal of Epidemiology*. 1999;150(4):327-333. doi:10.1093/oxfordjournals.aje.a010011
3. Rosenbaum PR, Silber JH. Using the Exterior Match to Compare Two Entwined Matched Control Groups. *The American Statistician*. 2013/05/01 2013;67(2):67-75. doi:10.1080/00031305.2013.769914
4. Silber JH, Rosenbaum PR, Clark AS, et al. Characteristics associated with differences in survival among black and white women with breast cancer. *JAMA*. Jul 24 2013;310(4):389-97. doi:10.1001/jama.2013.8272

© 2023 Chesley CF et al. *JAMA Network Open*.
