# Supplementary material for: Racial Disparities in Length of Stay Among Severely Ill Patients Presenting With Sepsis and Acute Respiratory Failure
Source: JAMA Netw Open. 2023 May 8;6(5):e239739. doi: 10.1001/jamanetworkopen.2023.9739 (PMC10167564; doi:10.1001/jamanetworkopen.2023.9739)
Supplement: Supplement 2. — Data Sharing Statement [file jamanetwopen-e239739-s002.pdf]

Data Sharing Statement

Chesley. Racial Disparities in Length of Stay Among Severely Ill Patients Presenting With Sepsis and Acute Respiratory Failure. *JAMA Netw Open*. Published May 08, 2023. doi:10.1001/jamanetworkopen.2023.9739

Data
Data available: No
